# Fluorocarbon Minimization Via Semifluorinated Copolymer Films by Combining Spin Coating and Ring-Opening Metathesis Polymerization

**DOI:** 10.1021/acs.langmuir.4c05253

**Published:** 2025-03-05

**Authors:** Matthew P. Vasuta, Zane J. Parkerson, Tyler D. Oddo, Bridget R. Rogers, G. Kane Jennings

**Affiliations:** † Interdisciplinary Materials Science Program, Vanderbilt University, Nashville, Tennessee 37235, United States; ‡ Department of Chemical and Biomolecular Engineering, Vanderbilt University, Nashville, Tennessee 37235, United States

## Abstract

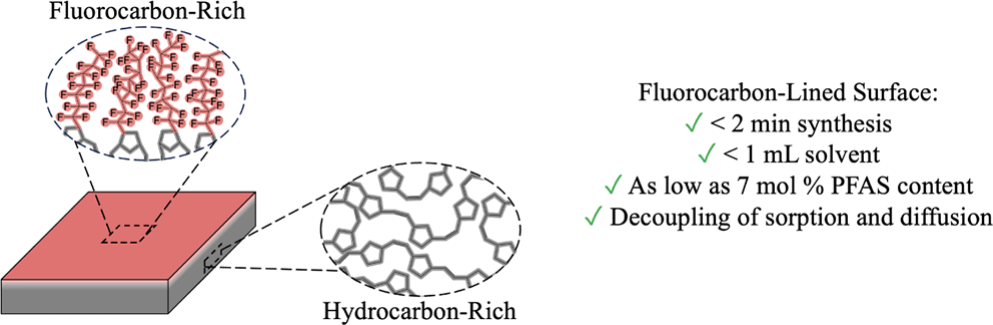

Per- and polyfluoroalkyl substances (PFAS) are ubiquitous in society largely due to their unique surface properties, but significant health concerns associated with these substances underscore the need for PFAS reduction strategies. We report a method to substantially reduce the amount of PFAS, solvent, and time needed to synthesize a low surface energy polymer film through the copolymerization of norbornene (NB) with 5-(perfluoro-*n*-alkyl)norbornenes (NBF*n*) in a single process that combines spin coating with ring-opening metathesis polymerization (scROMP). The unique scROMP approach efficiently integrates polymer film synthesis and deposition into one rapid process, converting monomer into polymer films in <2 min with <1 mL of solvent for a 36 cm^2^ film. Perfluoroalkyl chain lengths, *n*, of 4, 6, and 8 were examined, with the fluorocarbon component tending to dominate the surface for all n, exhibiting water contact angles comparable to those of the fluorocarbon homopolymer even with as little as 2% NBF*n* in the contacting monomer. As a potential application, these semifluorinated copolymer films were used in ethanol dehydration as low PFAS substitutes for amorphous fluoropolymer membranes. Even 7% fluorocarbon in the polymer (or 2% in the monomer) caused an order-of-magnitude increase in selectivity over a fully hydrocarbon membrane, with additional fluorination up to 63% (50% in monomer), leading to another order-of-magnitude enhancement and properties similar to the pNBF*n* homopolymer. Additionally, the dense outer fluorocarbon layer provided an ideal setup to estimate the sorption and diffusion components of selectivity for fluorocarbon and hydrocarbon groups within a membrane.

## Introduction

Fluorocarbon-based materials possess the lowest known surface energies of any functional materials, which is attributed to particularly weak intermolecular forces from the lack of polarizability and strong ionization potentials of C–F bonds.^1−3^ This low surface energy, combined with strong thermal stability, low flammability, chemical inertness, and low refractive index^4−6^ make thin films synthesized with perfluoromethyl (−CF_3_) and perfluoromethylene (−CF_2_−) groups highly stable in open air, aqueous, or organic environments and therefore desirable in numerous applications. However, concern is growing to limit the production of per- and polyfluoroalkyl substances (PFAS) containing −CF_3_ and −CF_2_– groups due to their potential impact on human health and the environment.^7−9^ Recent legislation has targeted the nonpolymeric PFAS monomers and plastic processing aids that are used during the synthesis of fluorinated polymers.^10−12^ Nonpolymeric PFAS lack the high stability and low solubility of polymeric PFAS, enabling these smaller molecules to accumulate within plant and animal tissue,^9,13^ which has been linked to cancer, kidney disease, altered thyroid and immune function, reproductive issues, and liver disease.^14,15^ To address these concerns and fulfill the need for fluorinated surfaces in many applications, films that possess these coveted surface properties but contain a minimum number of fluorinated functional groups must be developed.

**Scheme 1:**
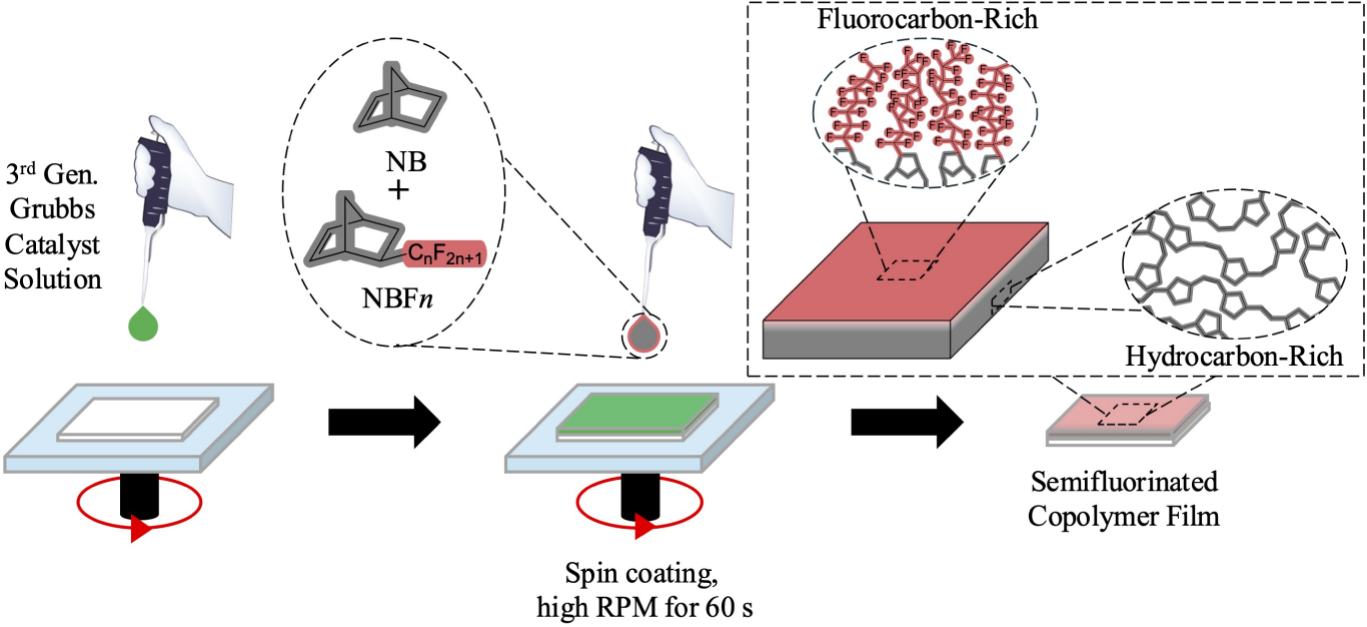
Simultaneous Spin Coating and Ring-Opening Metathesis Polymerization (scROMP) for the Rapid Synthesis of Semifluorinated Copolymer Films Norbornene (NB) is a fully hydrocarbon monomer, while 5-(perfluoro-*n*-alkyl)norbornenes (NBF*n*) are partially fluorinated monomers with perfluoro chains attached to norbornenyl rings.

Here, by designing copolymer films with controlled levels of fluorination, we seek to determine the minimum level of fluorocarbon needed to achieve the surface properties of a semifluorinated homopolymer film. We use the method spin coating ring-opening metathesis polymerization (scROMP), as shown in Scheme 1 that combines polymerization and film deposition into one process to rapidly synthesize copolymer films of high molecular weights and low polydispersities.^16^

Film growth can be modeled as a balance between radial spin-off of the monomer and upward growth of the polymer from the substrate, such that the polymer film thickness can be modified through a combination of spin speed, reaction time, and monomer concentration.^16^ The uniqueness of polymerizing while spin coating allows for fluorinated groups to migrate to the surface during polymerization, minimizing the amount of fluorinated substances needed to achieve the same surface properties as a semifluorinated homopolymer film.

Films with fluorocarbon-rich surfaces but minimal fluorinated substances in the bulk often leverage fluorocarbon-hydrocarbon immiscibility and surface energy minimization.^17^ In polymer systems with both fluorocarbon and hydrocarbon parts, individual fluorocarbon chains tend to strongly partition toward the surface and reorient themselves to minimize overall surface energy. −CF_3_ and −CF_2_– groups possess surface energies of 6 mJ m^–2^ and 18 mJ m^–2^, whereas −CH_3_ and −CH_2_– groups possess higher surface energies of 24 mJ m^–2^ and 31 mJ m^–2^,^1,2,18,19^ generating a thermodynamic driving force to localize fluorocarbon moieties on the surface of the film in air. Semifluorinated systems with ideally low surface energy contain both flexible polymer backbones and localized regions of high fluorination such that the fluorinated regions of the polymer self-associate, becoming incompatible with the hydrocarbon regions and placing their −CF_3_ and −CF_2_– groups along the surface.^20,21^

Most systems that maintain fluorocarbon surface energies with low fluorine-to-carbon atom ratios are within the category of side-chain fluorinated polymers, where either a select number of monomers can be fluorinated postpolymerization or a nonfluorinated monomer can be copolymerized with a monomer containing a fluorinated side chain.^22^ Post-polymerization fluorination can be an attractive route to generate semifluorinated films if control over the percentage of fluorinated monomer units can be achieved and the added fluorinated chain is long enough to generate fluoropolymer-level surface properties. Lewis et al. demonstrated the ability to improve the properties of polystyrene, several polycarbonates, and poly(ethylene terephthalate) through post-polymerization fluorination,^23^ while De Bruycker et al. observed water contact angles comparable to a fully fluorinated system at a 6% fluorinated triazolinedione repeat unit composition in the copolymer.^24^

Several groups have focused on copolymerizing a fluorinated monomer with a nonfluorinated, predominantly hydrocarbon monomer. These semifluorinated copolymerizations can be characterized by their polymer backbones, which tend to be polystyrenes,^21,25,26^ polyacrylates,^27−34^ polyurethanes,^35−37^ polyoxetanes,^38^ or polysilicones.^39−42^ Unfortunately, several of the above classes include copolymers that possess backbones that are too rigid or perfluoro side chains that are too short to properly segregate fluorocarbon groups to the surface. Polystyrenes and many polyacrylates are two such classes where the rigidity of the backbone hinders their ability to maintain fluorocarbon-level surface energies down to dilute levels of fluorination. Hopken and Moller’s fluorinated polystyrene and Ozbay and Erbil’s fluorinated polymethacrylate systems both show surface energy increases of >3.5 mJ m^–2^ as fluorination content is decreased from 25 to 10 mol %.^21,34^ Their systems contain glass transition temperatures (*T*_g_) that are >55 °C, suggesting that the rigidity of the polymers prohibits the localization of fluorocarbon moieties along the surface of the film. Saïdi et al. showed that the addition of more flexible alkyl acrylate monomers stabilizes the surface energy with decreasing levels of fluorination in the film, as they were able to maintain a surface energy of 12.0 mJ m^–2^ at only 5 mol % of the fluorinated repeat unit in the film.^31^ While the implementation of more flexible monomers can lower the surface energies of semifluorinated copolymer systems, a potentially more effective approach is to leverage the enhanced mobility of fluorocarbon monomers in the liquid phase before they become incorporated into the polymer state. Polymerizing while spin coating achieves this, as liquid fluorocarbon monomers are better able to migrate to the surface of the growing polymer film than polymerized fluorinated repeat units in traditional solution-based hydrocarbon-fluorocarbon copolymerization methods.

Norbornene-based monomers are ideal molecules for polymerizing while spin coating because they are rapidly polymerizable through ROMP and include both fluorinated and nonfluorinated analogues.^43−45^ We report the synthesis of poly(norbornene-*co*-(5-(perfluoro-*n-*alkyl)norbornene)) (p(NB-*co*-NBF*n*)) films with alkyl chain lengths of 4, 6, and 8 using scROMP on top of metal (gold), semiconductor (silicon), and porous polymer (polyacrylonitrile) substrates. Twenty-one of these copolymers were polymerized, each in under 2 min while spin coating, allowing for an orders-of-magnitude reduction in polymerization time and solvent from the traditional polymer film synthesis and deposition. The synthetic speed of scROMP to produce a wide range of overall fluorination and perfluoro chain lengths between copolymers enables rapid testing, evaluation, and materials discovery in a host of applications without the less sustainable requirement of bulk-scale polymer syntheses and separations.

One application of low surface energy films of particular interest to the authors is as thin film composite membranes for industrially relevant separations, specifically, the separation of water from ethanol. Current separation methods require extensive heating of liquid streams, which accounts for a staggering 10–15% of U.S. energy consumption.^46^ Separations performed using thin film composite membranes consisting of a dense polymer film on a porous support have shown great promise as energy-reducing alternatives, and amorphous perfluoropolymers are a class of membranes that have been demonstrated to successfully dehydrate organic solvents.^47−49^ The effect of reducing fluorination in ethanol dehydration, however, remains largely unstudied. In addition to assessing the compositional and surface properties of p(NB-*co*-NBF*n*) films, we performed scROMP of p(NB-*co*-NBF*n*)’s atop a porous polyacrylonitrile support and varied the amount of NBF*n* in different trials to determine how a reduction in PFAS affects ethanol dehydration performance, while enabling a decoupling of sorption versus diffusion selectivities.

## Experimental Section

### Materials and Methods

#### Materials

Silicon (100) wafers were purchased from University Wafers. Gold shot (99.99%) was purchased from J & J Materials. Poly(acrylonitrile) (PAN) supports (30 kDa cutoff) were obtained from Sterlitech. Grubbs catalyst second generation (1,3-bis(2,4,6-trimethylphenyl)-2-(imidazolidinylidene)(dichlorophenylmethylene)), 3-bromopyridine, hydroquinone, perfluoro(methylcyclohexane), *n*-hexane, diiodomethane, ethylene glycol, glycerol, and ethyl vinyl ether were purchased from Sigma-Aldrich. Norbornene (NB) (99%), dicyclopentadiene (DCPD) (96%), dichloromethane (DCM) (99.8%), *n*-octane (98+%), and potassium bromide (KBr) were used as received from Thermo Fisher Scientific. *n*-Pentane (98%), *n*-decane (99+%), *n*-dodecane (99%), *n*-tetradecane (99+%), *n*-hexadecane (99%), 1H,1H,2H-perfluoro-1-hexene (99%), 1H,1H,2H-perfluoro-1-octene (99%), and 1H,1H,2H-perfluoro-1-decene (99%) were purchased from Alfa Aesar. Hexane-d14 was purchased from Acros Organics. Ethanol (absolute) was used as received from Decon Laboratories. Deionized water (DI water) (16.7 MΩ·cm) was purified using a Modu-Pure system.

#### Preparation of Silicon Substrates

With the exception of silicon substrates prepared for profilometry in Figure S12, silicon (100) wafers were cut into 1.5 cm × 1.5 cm squares using a diamond scribe, rinsed with water and ethanol, and dried with a nitrogen gas stream prior to spin coating. Preparation of silicon substrates for Figure S12 followed the same procedure as above, except that silicon wafers were cut into 6 cm × 6 cm substrates to generate a larger distinction between the swollen and unswollen masses of the films.

#### Preparation of Gold Substrates

Chromium (100 Å) and gold (1250 Å) were sequentially evaporated onto silicon (100) wafers in a diffusion-pumped chamber at a rate of <2 Å/s. Gold-deposited wafers were cut into 1.5 cm × 1.5 cm squares using a diamond scribe, rinsed with water and ethanol, and dried with a nitrogen gas stream prior to spin coating.

#### Preparation of PAN Supports

PAN supports were cut using a razor blade into 6 cm × 6 cm squares and stored in a 90:10 v/v% mixture of deionized water and ethanol for 24 h to remove the glycerol preservative. The supports were then removed, rinsed with deionized water, and stored in a fresh solution of 90:10 v/v% deionized water and ethanol mixture until use.

#### Synthesis of NBF*n* Monomers

5-(Perfluorobutyl)norbornene (NBF4), 5-(perfluorohexyl)norbornene (NBF6), and 5-(perfluorooctyl)norbornene (NBF8) were synthesized via a Diels–Alder reaction as reported by Perez et al.^50^ For each synthesis, the 1H,1H,2H-perfluoro-1-alkene of appropriate chain length was combined in a Parr Instruments high-pressure reaction vessel with DCPD and hydroquinone at respective molar ratios of 1.9:1:0.03. Mixtures were heated in the vessel at 170 °C for 72 h and then purified by vacuum distillation. NBF*n* monomers were previously characterized by our group using ^1^H and ^19^F NMR with respective yields of 45%, 50%, and 43% for *n* = 4, 6, and 8.^44^

#### Polymerization

Grubbs catalyst third generation (G3) was synthesized from Grubbs catalyst second generation as reported by Love et al.^51^ To prepare the catalyst for polymerization, G3 was dissolved in DCM at a concentration of 5 mM for no more than 30 min before the polymerization.

Since NB is a solid at room temperature, NB monomer stock solutions were formulated to match the concentration of the neat fluorinated comonomer. For NBF4, the NB stock solution was 4.5 M, dissolved in pentane. Pentane was chosen as the solvent to reduce the viscosity of the monomer solution, making the solution easier to dispense for spin coating. With both the NB stock solution and the NBF4 neat monomer at 4.5 M, targeted comonomer solutions were prepared by combining the appropriate volumes of each in a vial and sonicating. All procedures were the same for NBF6 films, except the NB monomer stock solution was concentrated to 3.9 M to match the concentration of neat NBF6, and a 1:1 v/v% mixture of pentane and DCM was used as the solvent to assist in the miscibility of NBF6. NBF8 followed the same procedures as NBF6, but the NB stock solution needed to be concentrated to only 2.7 M to match the concentration of neat NBF8. For membrane tests on PAN and for films dissolved for nuclear magnetic resonance, only pentane was used as a solvent (the 1:1 v/v% mixture of pentane and DCM was not used) for the p(NB-*co*-NBF6) and p(NB-*co*-NBF8) systems.

Monomers were polymerized while spin coating using a SETCAS LLC KW-4A spin coater. Silicon or gold substrates were placed on a metal chuck with vacuum pulling from underneath. Only samples for X-ray photoelectron spectroscopy (XPS) and scanning electron microscopy with energy dispersive X-ray spectroscopy (SEM-EDS) were on gold substrates. Dispensing of catalyst and monomer occurred while spin coating as reported by Parkerson et al.^16^ First, 200 μL of 5 mM G3 solution was dispensed using a micropipette onto a spinning substrate at 2000 rpm for 30 s. After 30 s, the spin speed was increased, and 200 μL of a monomer or comonomer solution was dispensed and spin coated at 3000 rpm for 60 s. For the time study on the 50:1 NB:NBF8 films in Table S2, polymerization was quenched by spin coating 1 mL of ethyl vinyl ether to yield an inactive methyl-terminated polymer and Fischer carbene products. For the diffusional study in Figure S12, the spin speeds for the catalyst and monomer depositions were decreased to 1500 rpm to generate thicker films.

Films were synthesized on PAN supports for pervaporation testing. PAN supports tend to curl upon removal from DI water, so after drying with a stream of N_2_, the PAN supports were adhered to a silicon wafer using double-sided tape and spin coated. All other procedures used for Si and Au substrates were the same as for the PAN supports, except that 400 μL of catalyst solution and 400 μL of monomer solution were used instead of 200 μL solutions to account for the increased 36 cm^2^ area of the PAN support relative to the Au or Si substrates.

#### KBr Pellet Formation

The KBr pellet method was used for sample preparation for transmission infrared spectroscopy. Films were scraped off the surface of the silicon substrates using a razor blade and ground with 200 mg of KBr in a mortar and pestle. The resulting mixture was compressed in a Specac 13 mm die at 7.5 tons of force for 10 min using a Specac Atlas 15T manual hydraulic press.

#### Dissolution of Polymer Films for ^1^H NMR

For NMR characterization, polymer films were removed from silicon substrates using a razor blade and dissolved in 700 μL of the appropriate solvent. Films of NB or monomer composition from 100:1 NB:NBF*n* to 3:1 NB:NBF*n* dissolved after 1 h of stirring at room temperature. Films that were 1:1 NB:NBF*n*, 1:3 NB:NBF*n*, or NBF*n* required 24 h of stirring at 60 °C to fully dissolve.

For Figure S1, NB and NB:NBF4 copolymers with ratios from 100:1 NB:NBF4 through 1:1 NB:NBF4 spectra were acquired on the 400 MHz spectrometer using 16 scans and CDCl_3_ as the solvent. 1:3 NB:NBF4 and NBF4 spectra were collected using the 600 MHz spectrometer with 64 scans and CDCl_3_ as the solvent. For Figure S2, NB and NB:NBF6 copolymers with ratios of 100:1 NB:NBF6 through 3:1 NB:NBF6 spectra were acquired on the 400 MHz spectrometer using 16 scans and CDCl_3_ as the solvent. 1:1 NB:NBF6, 1:3 NB:NBF6, and NBF6 spectra were acquired using the 600 MHz spectrometer with 64 scans and CDCl_3_ as the solvent. For Figure S3, NB and copolymers with ratios 100:1 NB:NBF8 through 10:1 NB:NBF8 spectra were acquired on the 600 MHz spectrometer using 16 scans and a CDCl_3_ solvent. 3:1 NB:NBF8 and 1:1 NB:NBF8 spectra were collected with the 600 MHz spectrometer using 64 scans and CDCl_3_ as the solvent. The 1:3 NB:NBF8 and NBF8 spectra were gathered using the 600 MHz spectrometer with 64 scans and 600 μL of perfluoro(methylcyclohexane) and 100 μL of hexane-d14 as cosolvents.

### Characterization Techniques

Transmission infrared spectroscopy was performed using a Thermo Fisher Scientific Nicolet 6700 FTIR instrument with a DTGS KBr detector. Each spectrum was gathered using 64 scans with a blank KBr pellet as a background and analyzed using OMNIC software.

Attenuated total reflectance Fourier transform infrared spectroscopy (ATR-FTIR) was performed on a Thermo Fisher Nicolet 6700 FT-IR spectrometer with a liquid nitrogen-cooled mercury-cadmium-telluride detector and Smart iTR ATR attachment with a diamond-crystal plate. Spectra of samples were collected from 4000–400 cm^–1^ through 128 scans at a 2 cm^–1^ resolution.

Nuclear magnetic resonance (NMR) experiments were acquired on a 9.3 T Bruker magnet equipped with a Bruker AV console operating at 400.13 MHz and a 14.0 T Bruker magnet equipped with a Bruker AV-111 console operating at 600.13 MHz. Experimental conditions included 32,000 data points, a 13 ppm sweep width, and a recycle delay of 1.5 s.

Gel permeation chromatography (GPC) of pNB and select p(NB-*co*-NBF4) films synthesized by scROMP was completed in a previous study, showing polydispersities of ∼1.2 and number-averaged molecular weights >200 kDa.^31^ Additional GPC was not performed in this study as the p(NB-*co*-NBF*n*) films with *n* = 6 and 8 are insoluble in any of the common solvents used for GPC.

Contact angle goniometry measurements were obtained using a Ramé-Hart manual goniometer with drop sizes of ∼5 μL of water or hexadecane. The dispensing syringe remained inside the droplets for advancing and receding contact angles.

Differential scanning calorimetry (DSC) data was collected on a TA Instruments DSC 25 with a Refrigerated Cooling System 40. Polymer films were scraped off gold surfaces using a razor blade and heated under the following regimen: (1) 25 to 200 °C at 10 °C/min, (2) 200 to −20 °C at −10 °C/min, and (3) −20 to 200 °C at 10 °C/min. Polymer films were held at a constant temperature for 10 min between cycles, and reported DSC curves and glass transition temperatures were taken from the second heating cycle.

X-ray photoelectron spectroscopy (XPS) was performed using an Ulvac-PHI Versaprobe 5000. Monochromatic Al Κα X-rays (1486 eV) from a 100 μm diameter X-ray beam were rastered over an ∼1000 μm by 500 μm area in each acquisition. Take-off angles of 30°, 45°, or 90° from the sample surface were used to control the information depth of the analysis. Pass energies of 187.7 and 23.5 eV were used for the survey and high-resolution acquisitions, respectively. Charge neutralization was accomplished using 1.2 eV electrons and 10 eV Ar^+^ ions. Binding energies were calibrated to −CH_2_– type bonding in the carbon 1s spectrum at 284.8 eV.

Scanning electron microscopy (SEM) images were gathered using a Zeiss Merlin scanning electron microscope with an Everhart–Thornley secondary electron detector operating at a beam energy of 5 kV and a working distance of 8.5 mm. Samples were attached at a 90° angle to an SEM stub using double-sided carbon tape. Energy dispersive X-ray spectroscopy (EDS) spectra were gathered with an Oxford Instruments Silicon Drift Detector and analyzed using Aztec software.

Pervaporation tests were completed using a Sterlitech Polytetrafluoroethylene Innovator tangential flow cell with a 16 cm^2^ active area. Feed solutions were heated to 60 °C and flowed to the membrane interface using a rotary pump. Pervaporation was achieved using an Edwards 5 vacuum pump. Permeate was condensed and collected using a liquid nitrogen-cooled cold trap, and ethanol content was measured using an Atago PAL-34S pocket refractometer. All membranes tested were synthesized on PAN supports with a 36 cm^2^ surface area.

Profilometry analysis was performed on a Veeco Dektak 150 contact stylus profilometer with a 12.5 μm stylus tip radius and an applied force of 29.4 μN. Scans were 1 mm long, and films were scratched with tweezers to reveal the bare Si substrate as a baseline for film thickness comparison.

## Results and Discussion

Twenty-one different p(NB-*co*-NBF*n*) films were synthesized by varying NB:NBF*n* ratios and perfluoro chain length *n* to assess how modifying the degree and localization of fluorination affects a film’s surface properties and ability to dehydrate ethanol by pervaporation. All films were synthesized by the scROMP process in <2 min using <1 mL of solvent per film, demonstrating a fast and low-waste method to produce copolymer films and membrane selective layers with ultralow surface energies and controlled levels of fluorination. scROMP has been shown to produce polymers and copolymers with high molecular weights (400–700 kDa) and low polydispersities (<1.2).^16^

### Film Composition

**Figure 1:**
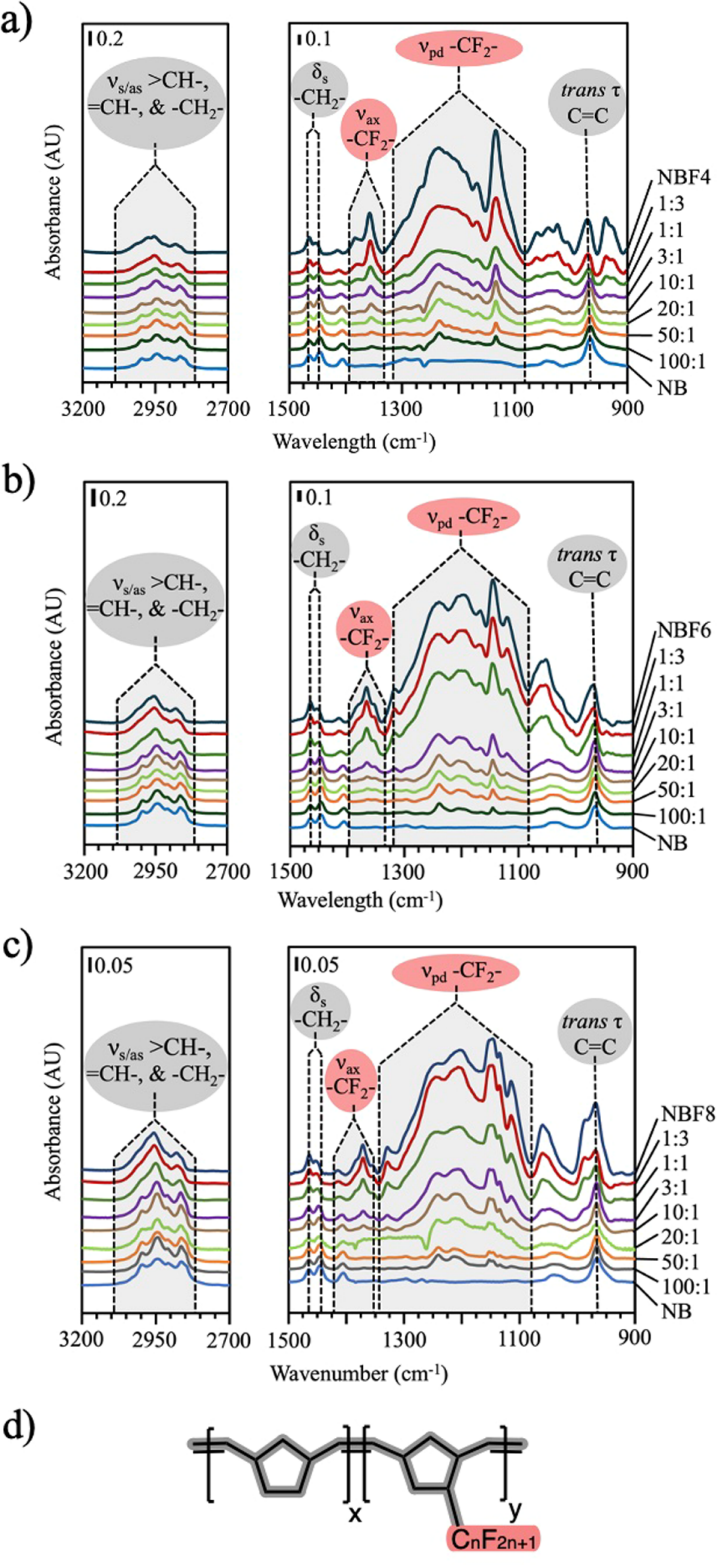
Transmission IR spectra of p(NB-*co*-NBF*n*) copolymer films with pNB and pNBF*n* homopolymer films. (a) pNB, p(NB-*co*-NBF4), and pNBF4. (b) pNB, p(NB-*co*-NBF6), and pNBF6. (c) pNB, p(NB-*co*-NBF8), and pNBF8. (d) Chemical structure of p(NB-*co*-NBF*n*) films. Fluorocarbon moieties are shown in red, hydrocarbon moieties are shown in gray, monomer ratios are listed as molar ratios of NB to NBF*n*, and all spectra are normalized so that the C–H stretching regions are equivalent areas.

Transmission IR was used to obtain compositional data on p(NB-*co*-NBF*n*) films generated by using scROMP. Figure 1 shows transmission IR spectra of the homopolymer films of pNB and pNBF*n* along with p(NB-*co*-NBF*n*) films at NB:NBF*n* reagent ratios from 1:3 to 100:1 for *n* = 4, 6, and 8.

Peaks for methine and methylene stretching (υ_s/as_ >CH–, =CH–, and −CH_2_– (3050–2800 cm^–1^)), methylene scissoring (δ_s_ −CH_2_– (1464 and 1446 cm^–1^)), and *trans* olefin out-of-plane bending (*trans* τ C=C (971 cm^–1^)) are all more prominent in films synthesized with higher NB concentrations. In contrast, axial perfluoromethylene stretching (υ_ax_ −CF_2_– (1400–1340 cm^–1^)) and perpendicular perfluoromethylene stretching (υ_pd_ −CF_2_– (1320–1080 cm^–1^)) regions^52^ increase for films prepared with increasing NBF*n* concentration. These IR spectra indicate that the composition of the copolymer film can be varied by changing the ratio of the two reagents.

**Figure 2:**
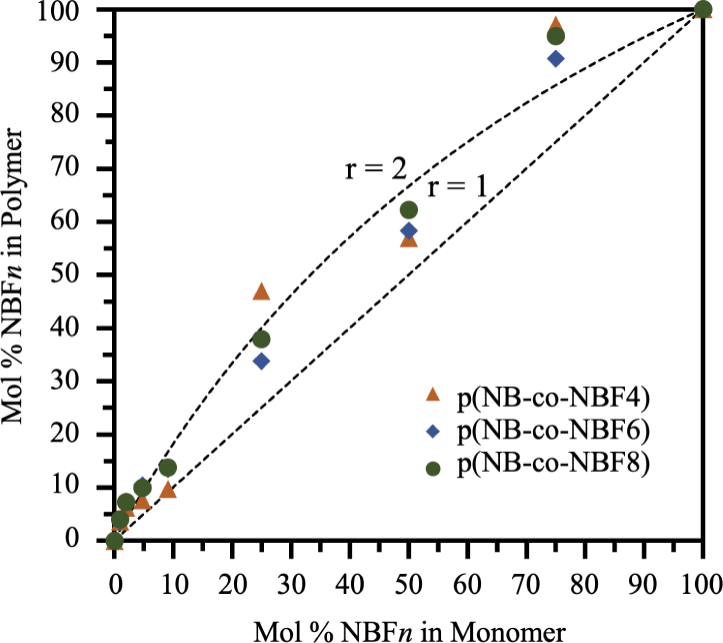
Mol % NBF*n* in polymer vs mol % NBF*n* in the monomer solution. Mol % NBF*n* in the polymer is calculated from ^1^H NMR of the bulk polymer film. Incorporation ratios greater than 1 appear above the 45° line and indicate a more favorable incorporation of NBF*n* over NB in the polymer. Dashed lines and curves show example theoretical values if *r* = 1 or *r* = 2.

To quantify the relative incorporation of monomers in each polymer film, ^1^H NMR data in Figures S1–S3 were used to determine the bulk composition of the p(NB-*co*-NBF*n*) films as reported in Figure 2. Solvent-only NMR spectra, labeled repeat unit protons, and tabulated NMR integration values are located in Figures S4 and S5, and Table S1, respectively. The incorporation ratio, *r*, defined as
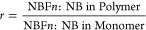
1is used to assess the relative incorporation of two different monomers into the structure of the polymer, with *r* > 1 demonstrating a greater molar incorporation of NBF*n* than NB into the polymer film. Figure 2 indicates a more favorable incorporation of the NBF*n* monomer over NB regardless of the perfluoro chain length.

In addition, a time study of the 50:1 NB:NBF8 film composition in Table S2 shows that the incorporation of the NBF8 monomer doubles as the polymerization time increases from 10 to 60 s. Collectively, these results suggest that (1) the NBF*n* monomer is better entrained in the growing film with lower fractional spin-off versus that of NB, and (2) the NBF*n* monomer becomes enriched near the outer interface of the growing film to minimize interfacial free energy,^53^ and is thus increasingly incorporated at longer polymerization times.

### Surface Properties

Figure S6 shows a Zisman plot that identifies the critical surface tension (γ_c_) for the pNBF4, pNBF6, and pNBF8 homopolymers as 18, 16, and 8 mJ m^–2^, respectively, which are comparable to the critical surface tensions of pNBF*n* films gathered by Faulkner et al. using surface-initiated ROMP (siROMP) of 19, 13, and 9 mJ m^–2^ for *n* = 4, 6, and 8.^44^ The γ_c_ values for the pNBF*n* films indicate fluorocarbon-dominated surfaces, with pNBF4 suggesting −CF_2_– dominance, pNBF6 showing a mixture of mostly −CF_2_– with some −CF_3_, and pNBF8 showing −CF_3_ dominance. These surface energies are considerably lower than the −CH_2_– rich pNB film, which has a γ_s_ = 37 mJ m^–2^ from the Owens–Wendt plot in Figure S7 (γ_s_ ≈ γ_c_, in this case). Contact angle goniometry was used to assess changes in surface properties of p(NB-*co*-NBF*n*) films as the amount of fluorination in the system is decreased. We have used water and hexadecane as probe liquids for their ability to distinguish −CF_3_ and −CF_2_– surface compositions present in the pNBF*n* films from −CH_2_– compositions that dominate for pNB. If a surface is flat and homogeneous, advancing water and hexadecane contact angles are expected to be near 118° and 79° for −CF_3_,^54^ 109° and 45° for −CF_2_–,^55,56^ and 96° and <10° for −CH_2_–,^57,58^ respectively.

**Figure 3:**
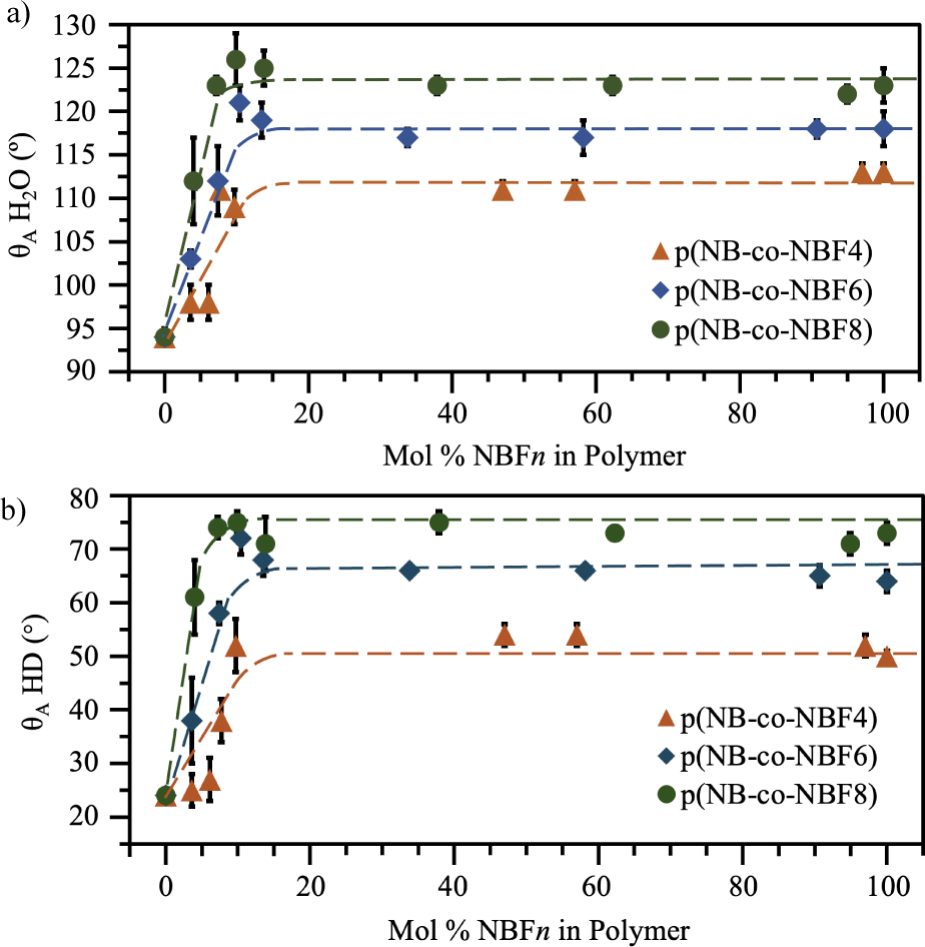
(a) Advancing water contact angles and (b) “initial” advancing hexadecane contact angles versus mol % NBF*n* in the bulk polymer film for p(NB-*co*-NBF4), p(NB-*co*-NBF6), and p(NB-*co*-NBF8) films. Dotted lines are intended to serve as guides for the eye. If an error bar is not visible, then the error is represented by the size of the symbol.

Water contact angles for the p(NB-*co*-NBF*n*) systems are shown in Figure 3a. Comparing the homopolymer films at 0% and 100% on the *x*-axis for pNB and pNBF*n*, respectively, pNBF4 shows water contact angles that are 18° higher than those of pNB, suggesting the presence of −CF_3_ and −CF_2_– groups on the pNBF4 surface. Water contact angles remain approximately the same as those for pNBF4 in copolymer compositions down to 8% NBF4 in the bulk film, indicating that the polymer chains place the low-energy perfluoro chains at the surface, even at low concentrations of pNBF4 in the copolymer film. This result is consistent with other reports in the literature, as unfavorable interactions between the fluorocarbon side chains and the hydrocarbon backbone cause localized surface arrangement of the fluorocarbon chains.^17,20,21,59^ The additional perfluorinated carbons in pNBF6 and pNBF8 increase water contact angles by 6° and 10° relative to pNBF4, which is attributed to a greater energetic driving force to expel fluorinated chains of greater length to the surface.^17,21^ More importantly, water contact angles for the copolymer films remain approximately the same as those for the pNBF6 and pNBF8 homopolymers for down to 10% NBF6 and 7% NBF8 in the bulk film, respectively.

Hexadecane contact angles were measured in addition to water contact angles, as hexadecane can better distinguish between the dispersive components of hydrocarbons and fluorocarbons.^1,2,18,60^Figure 3b shows the advancing hexadecane contact angles when the droplet was allowed to “initially” touch the surface while still being connected to the needle. These “initial” advancing hexadecane contact angles suggest similar surface properties of the copolymer films to those interpreted from the water contact angles. Contact angles are 52°, 63°, and 73° for homopolymers of pNBF4, pNBF6, and pNBF8, which are relatively consistent with the −CF_3_- and −CF_2_–dominated surfaces. Hexadecane contact angles are also maintained down to 10, 10, and 7% fluorination for the p(NB-*co*-NBF4), p(NB-*co*-NBF6), and p(NB-*co*-NBF8) systems, respectively. Both water and hexadecane contact angles remain stable with exposure time, as shown by the maintenance of contact angle with time shown in Table S3. Additionally, these systems represent a drastic reduction in PFAS usage while still maintaining a fluorocarbon surface, with estimates in Table S4 showing an ∼20× decrease in moles of PFAS used and an ∼50× decrease in volume of PFAS-enriched waste over a traditional method for synthesizing a polymer film with a fluorocarbon surface.

**Figure 4:**
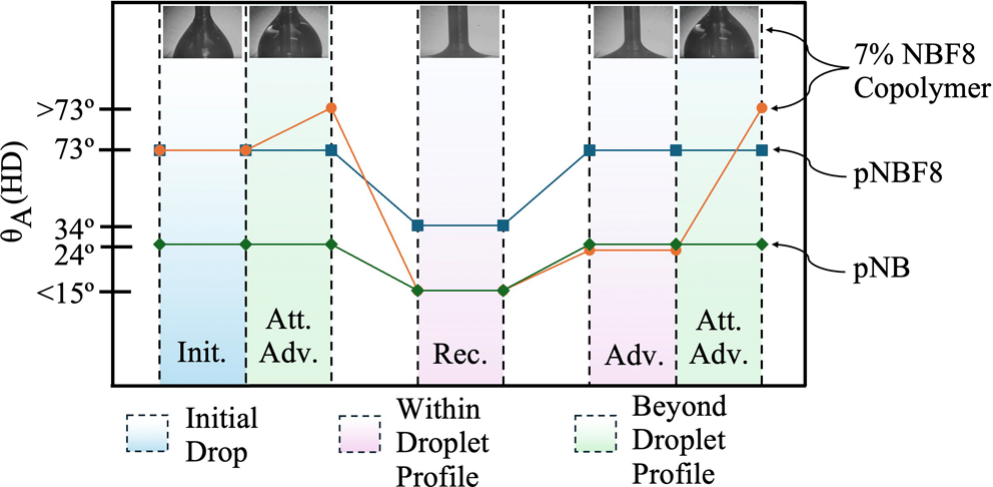
Advancing and receding hexadecane contact angles on 7% NBF8 copolymer, pNBF8, and pNB films. Note that both pNB and pNBF8 advance and recede as expected. The 7% NBF8 copolymer displays the following behavior when attempting to advance and recede: (init.) initial contact angle when hexadecane just touches surface and the needle is inside the droplet; (att. adv.) swelling of the nonadvancing droplet when attempting to further advance; (rec.) receding contact angle; (adv.) immediate advance of the droplet within the already prewet area after receding; (att. adv.) swelling of the droplet when attempting to further advance beyond the prewet area.

Interestingly, in cases with dilute fluorocarbon for contact angles that are comparable to those of the homopolymer, the hexadecane droplet is unable to advance beyond initial contact with the surface. A contact angle goniometer experiment was performed on a 7% NBF8 copolymer film to further understand these phenomena, as shown in Figure 4.

The “initial” column in Figure 4 shows the droplet as soon as the needle is extended downward and contacts the surface. The contact angle is 73°, consistent with the mostly −CF_3_ dominated surface of a pNBF8 homopolymer film. After the initial contact of hexadecane with the surface, however, the inability to advance the droplet beyond its initial size in the first attempted advancing column suggests a dramatic change in surface energy on the outside edge of the drop versus that on the inside edge. We hypothesize that the −CF_3_ and −CF_2_– groups that compose the surface in air reorient away from the surface underneath the hexadecane droplet to expose hydrocarbon groups that exhibit more favorable interactions with the probe liquid. Since the area under the drop is now hydrocarbon and the area outside the drop is still fluorocarbon, advancing the droplet beyond its initial contact would be energetically unfavorable. This theory is further supported when the droplet is receded, where instead of showing a higher receding contact angle of 34° like the pNBF8 homopolymer, the contact angle is <15°, consistent with a pNB homopolymer surface. If the droplet is then advanced again within a few seconds of receding and within the bounds of the original droplet, then the advancing contact angle is also consistent with a pNB −CH_2_– dominated surface. Once the advance reaches the boundary of the original droplet, however, the hexadecane probe liquid can no longer advance. The combination of water and hexadecane contact angles supports that the 7% NBF8 copolymer shows a fluorocarbon-rich surface energy of (∼8 mJ m^–2^) in open air and aqueous environments but switches to a hydrocarbon surface (∼37 mJ m^–2^) in hydrocarbon environments.

Contact angles were previously used to show hydrocarbon-fluorocarbon surface rearrangement in several fluorinated acrylate systems.^61−65^ Surface rearrangement has been observed only in acrylate polymers without bulky groups, with fluorocarbon chain lengths of *n* ≤ 6 at room temperature. Side chain crystallization is often observed for chain lengths *n* ≥ 8, making longer perfluoro chain analogues show surface rearrangement only at elevated temperatures.^61,63^ Our dilute fluorocarbon p(NB-*co*-NBF*n*) systems are distinct from the above fluorinated acrylate systems by their ability to demonstrate surface rearrangement at chain lengths of *n* = 4, 6, and 8 at room temperature. DSC scans in Figure S8 do not show melting peaks characteristic of side-chain crystallization in the dilute fluorocarbon p(NB-*co*-NBF*n*)’s for any perfluoro chain length or the homopolymer pNBF8. These absences of melting peaks suggest that our p(NB-*co*-NBF8) systems do not form as rigid fluorocarbon layers at the surface as do fluorinated polyacrylates with *n* = 8 and can more readily reorient surface groups in response to a change in stimuli.

### Surface Composition

Contact angle goniometry is known to probe only the outer few Å of the film.^66^ To probe further into the depth of the film, X-ray photoelectron spectroscopy was used to assess the composition of the outer ∼5 nm of the film. Since the pNBF8 homopolymer film and the 7% NBF8 copolymer film both showed fluorocarbon-rich surfaces in contact angle goniometry, XPS was used as an additional technique to verify the presence of a dense fluorocarbon layer and to provide an estimate of the thickness of the layer.

**Figure 5:**
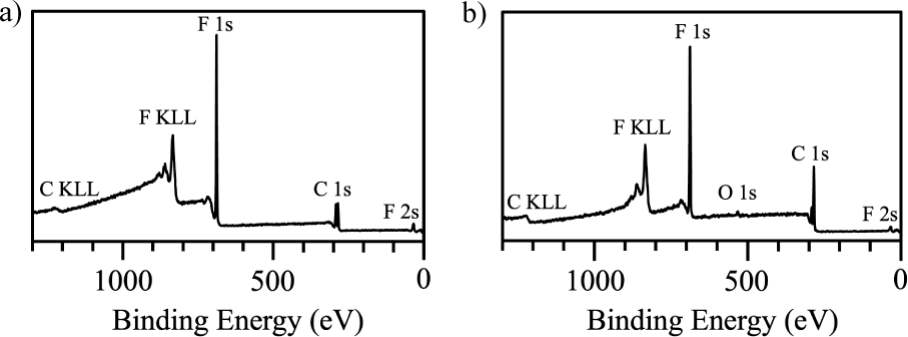
XPS survey spectra obtained from films of (a) pNBF8 and (b) 7% NBF8 copolymer.

Figure 5 displays XPS survey spectra for films of the homopolymer pNBF8 and the 7% NBF8 copolymer, and Figure S9 shows a survey spectrum for a pNB film. The survey spectra of Figure 5 show the dominant and expected contributions of F and C in the film. A small signal for O at 531 eV in the copolymer is attributed to slight oxidation of some of the olefinic bonds in the pNB backbone^67^ or adventitious hydrocarbons from atmospheric exposure. pNBF8 demonstrates the presence of fluorocarbon through binding peaks for F 1*s* at 689 eV, F 2*s* at 30 eV, and C 1*s* at 286 eV. The 7% NBF8 copolymer shows the same peaks as pNBF8 but exhibits a stronger C 1*s* peak from increased hydrocarbon content.

**Figure 6:**
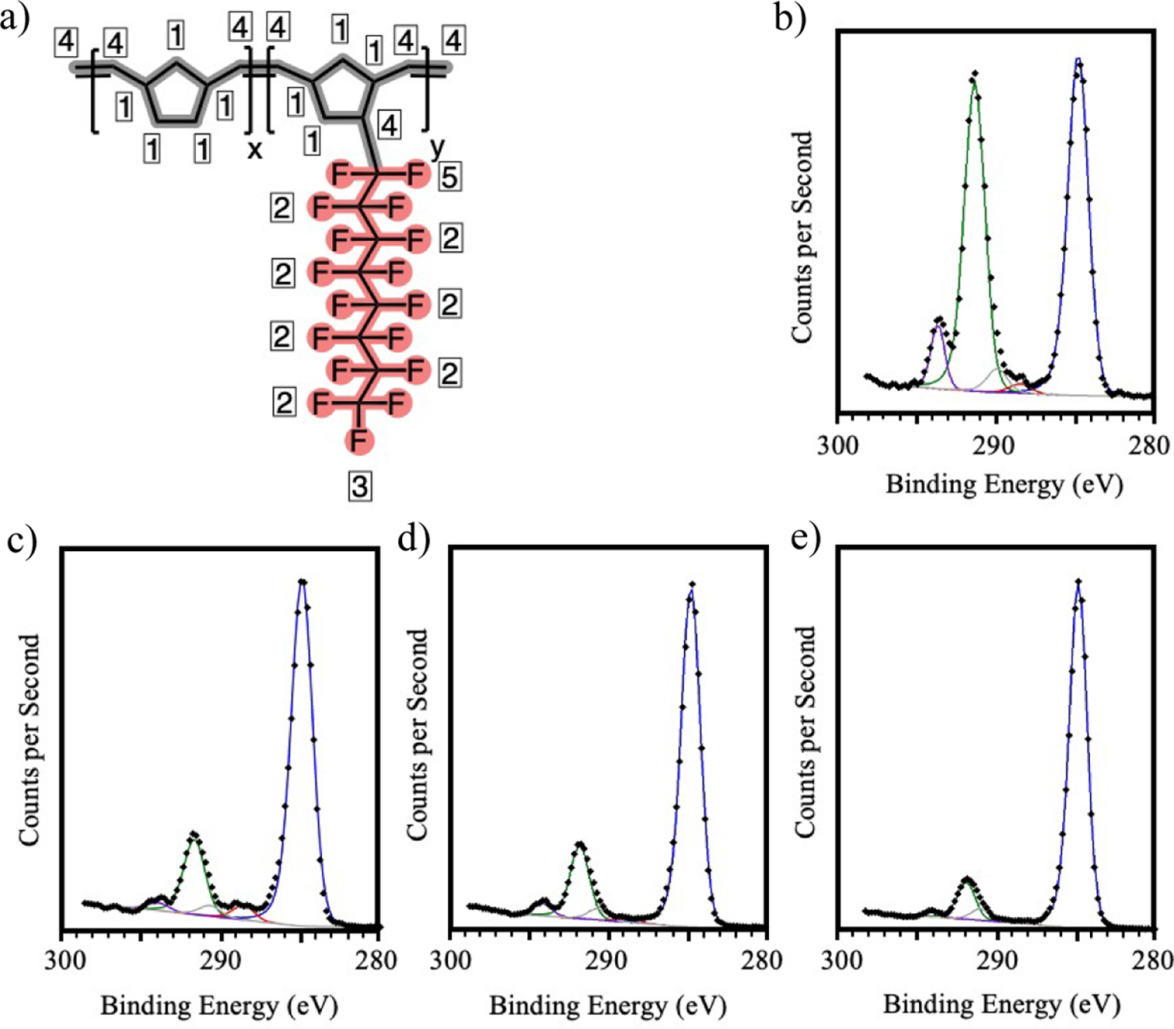
(a) Sketch of the molecular structure of the 7% NBF8 copolymer. Boxed numbers indicate the different carbon bonding environments of similar bonding energy: 1 = C–C (blue), 2 = C–F_2_ (green), 3 = C–F_3_ (purple), 4 = C*-CF_2_ and C = C (red), 5 = C–C*F_2_ (gray). (b) XPS C 1*s* spectrum acquired using a 45° takeoff angle from a pNBF8 film. XPS C 1*s* spectrum acquired using a (c) 30° takeoff angle, (d) 45° takeoff angle, and (e) 90° takeoff angle from a 7% NBF8 copolymer.

High-resolution spectra showing C 1*s* peak deconvolution for pNBF8 and the 7% NBF8 copolymer films are presented in Figure 6. Figure 6a shows five distinct bonding environments for the C 1*s* peak, which appear at 294 eV for C–F_3_, 292 eV for C–F_2_, 291 eV for C–C*F_2_, 289 eV for C*-CF_2_ and C=C, and 285 eV for C–C in Figure 6b for the pNBF8. Figure 6d shows the C 1s region for the 7% NBF8 copolymer spectrum obtained at the same 45° takeoff angle as the spectrum for pNBF8 in Figure 6b, which was estimated in the Section S6 to correspond to the outer ∼3.2 nm of the film. When compared to the pNBF8 spectrum, the peaks assigned to fluorocarbon in the 7% NBF8 copolymer are reduced relative to the C–C peak, which is expected given the overall reduction of fluorination in the film. Table 1 quantifies this difference, showing that the F:C atom ratio is only 0.65 in the 7% NBF8 copolymer compared to 1.11 in the pNBF8 homopolymer.

**Table 1:** Fluorine-to-Carbon Atom Ratio and Distribution of Carbon Bonding in pNBF8 and 7% NBF8 Copolymer Films Determined Using XPS at Different Take-off Angles

	pNBF8	7% NBF8 Copolymer		
Take-off Angle (deg)	45	30	45	90
F:C Atom Ratio	1.11	0.63	0.63	0.46
*Fraction of Carbon Bonding*				
C–C	0.46	0.78	0.78	0.88
C–F_2_	0.43	0.17	0.17	0.99
C–F_3_	0.06	0.03	0.03	0.01
C*-CF_2_ and C=C	0.01	0.02	0.01	0.01
C–C*F_2_	0.03	0.01	0.01	0.01

Nonetheless, a comparison of these numbers suggests that the concentration of NBF8 repeat units in the copolymer is well above 7% in this outer surface region probed by XPS. To determine if the F:C ratio increases with greater surface sensitivity, the takeoff angle was reduced to 30° in Figure 6c. Sampling closer to the surface by reducing the probe depth below ∼2.3 nm revealed no increases in % fluorination. However, increasing the takeoff angle and probe depth to 90° and ∼4.5 nm significantly reduced the peaks assigned to C–F relative to those assigned to the C–C peak from an F:C ratio of 0.65 to 0.46, indicating that fluorination decreases at depths greater than ∼3.2 nm from the surface. The fluorocarbon-rich outer region of the 7% NBF8 copolymer is then estimated to be no thicker than ∼3.2 nm. Combining contact angle and XPS data suggests that the outer 0.5 nm of the 7% NBF8 copolymer is exclusively fluorocarbon, while the outer ∼3.2 nm is fluorocarbon-enriched relative to the remaining depth of the film.

**Figure 7:**
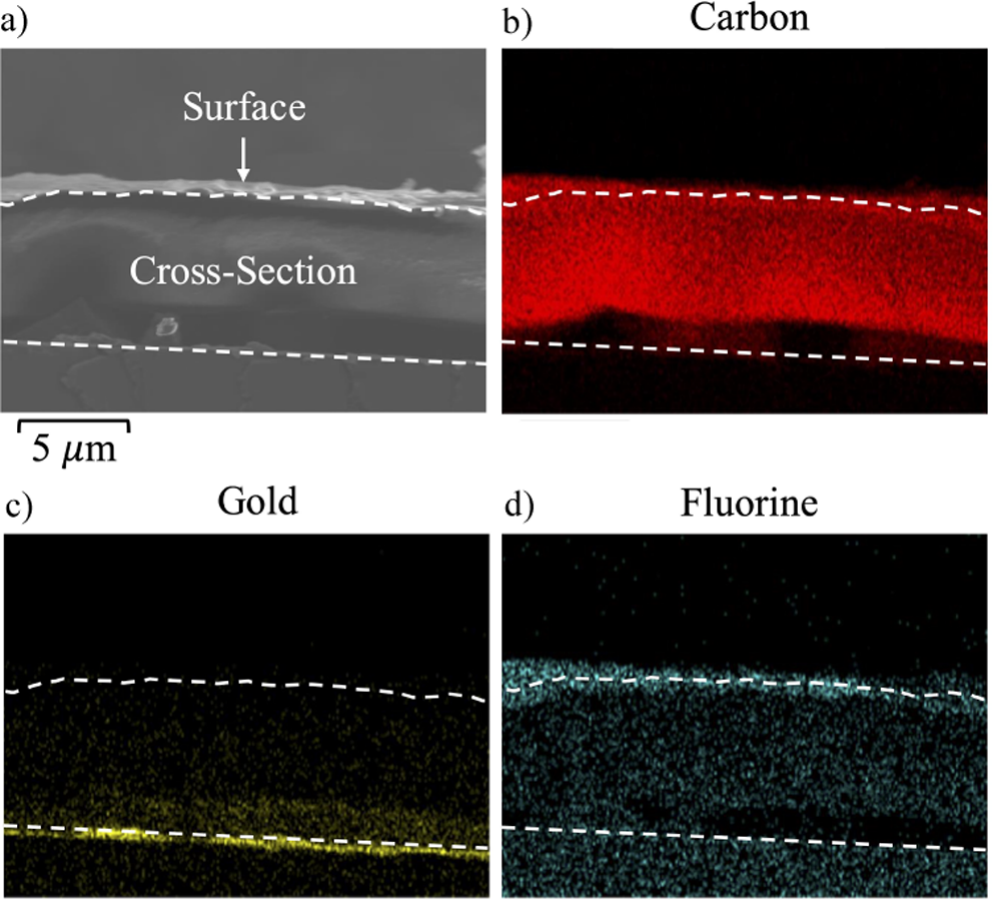
(a) SEM image of a cross section of a 7% NBF8 copolymer film synthesized on top of a gold substrate. Energy dispersive X-ray spectroscopy maps for (b) carbon, (c) gold, and (d) fluorine. Dashed lines are drawn as guides to the eye to separate the surface of the film from the cross-section and the substrate.

SEM-EDS was used as a visual technique to examine the presence of a fluorocarbon throughout the cross-section of a copolymer film grown atop a gold substrate. Figure 7 shows a cross-sectional SEM-EDS image of a 7% NBF8 copolymer with elemental maps for carbon, gold, and fluorine. Dashed lines indicate the positions of the surface (top) and the gold substrate (bottom) for all figures, as supported by the enhanced signal for the gold layer in Figure 7c, setting the bottom limit of the copolymer film. Above the gold, there is a sharp transition to a predominantly carbon region of this several micron-thick film, which terminates with a thin fluorocarbon-rich layer near the surface. Figure S10 includes an EDS spectrum showing the relative abundance of elements within the cross-section in Figure 7.

### Ethanol Dehydration

**Figure 8:**
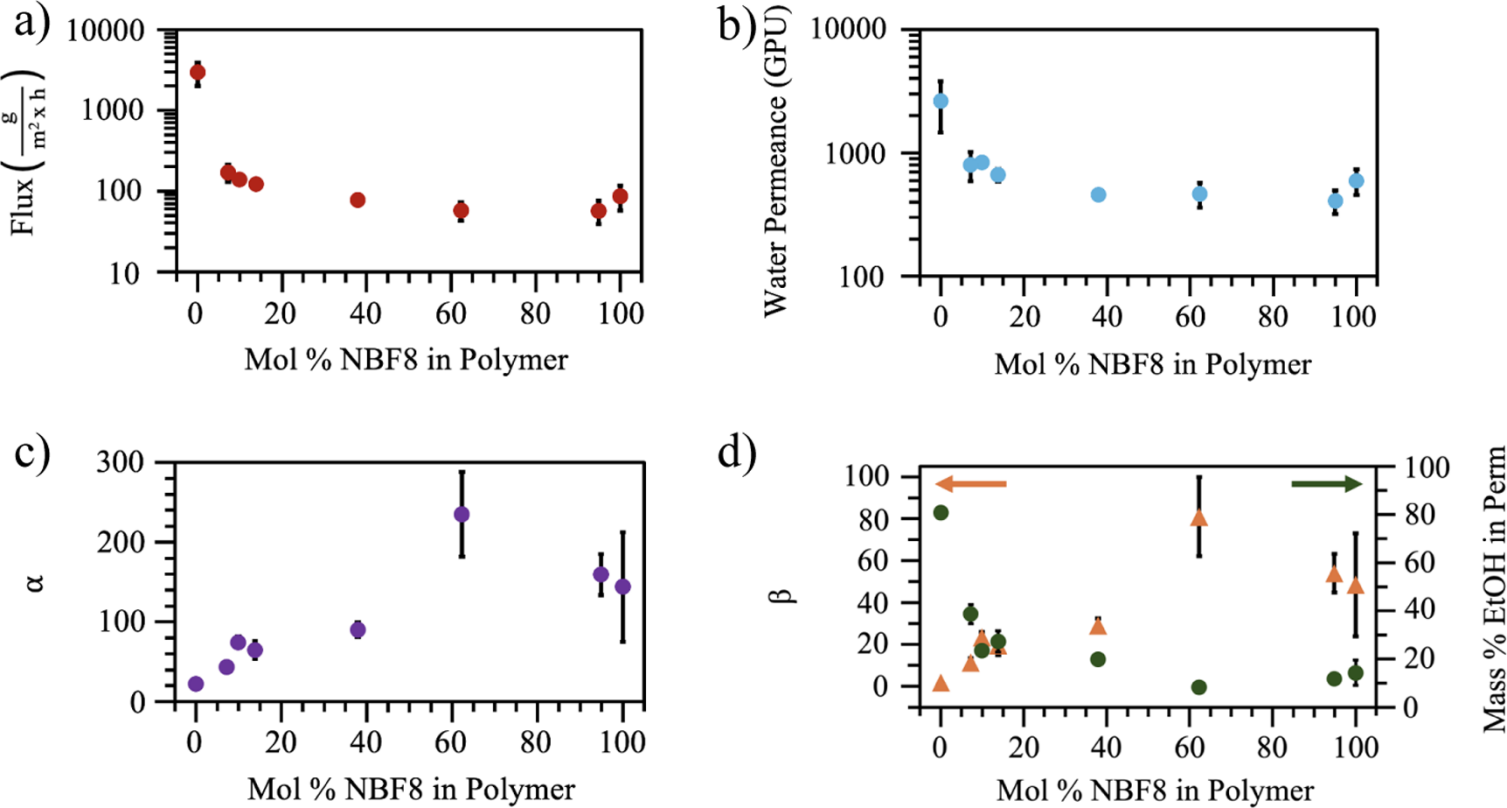
(a) Flux, (b) water permeance, (c) selectivity, and (d) separation factor and mass % ethanol in the permeate of p(NB-*co*-NBF8) membranes as mol % NBF8 in the polymer membrane is increased. One gas permeation unit (GPU) is equivalent to 10^–6^ cm^3^ (STP)·cm^–2^·cm Hg^–1^·s^–1^. The initial feed solution was 90 mass % ethanol. If an error bar is not visible, the error is represented by the size of the symbol.

Since amorphous perfluoropolymer membranes with high PFAS content have performed well in ethanol dehydration studies,^48,49^ we sought to explore the effect of fluorocarbon content in p(NB-*co*-NBF*n*) films and the presence of a dense fluorocarbon outer surface on performance in ethanol dehydration. The presence of a dense fluorocarbon layer has been previously shown to limit excess swelling of the film, which often leads to poor membrane performance.^48^ Additionally, fluorinated membranes are known to possess large fractional free volumes, which have been demonstrated to leverage the diffusional advantage that smaller water molecules have over larger ethanol molecules when permeating through a membrane.^68^

p(NB-*co*-NBF8) membranes were synthesized on PAN supports and placed in a tangential flow cell for pervaporation, with one side exposed to a 90/10 mass ratio of ethanol/water at 60 °C and the opposing side exposed to a vacuum. All polymer membranes were assumed to be in the rubbery state based on DSC analysis in Section S5. Figure 8 shows the total flux of liquid across the membrane, the water permeance, the selectivity of water over ethanol across the membrane, the separation factor of water over ethanol across the membrane, and the percentage of ethanol in the accumulated liquid that permeated across the membrane.

Selectivity (α) is defined as
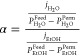
2where *j*_*i*_ is the component molar flux,  is the component vapor pressure in the feed stream, and  is the component vapor pressure in the permeate stream. The separation factor (β) is a ratio of permeate and feed compositions that does not account for the thermodynamics of the feed and permeate mixtures, and is defined as
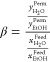
3where  is the component vapor mole fraction in the permeate and  is the component liquid mole fraction in the feed. Ideal systems would show high water fluxes and values for α and β that are far greater than 1 to demonstrate that water was selectively pulled from the ethanol-rich feed and accumulated in the permeate. To describe the transport of chemical species through a given polymeric membrane, the solution-diffusion model is employed,^72^ and the selectivity can be written as
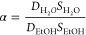
4where *D*_*i*_ and *S*_*i*_ are the diffusivity and solubility of feed molecules in the membrane.

Figure 8 shows that pNB is largely nonselective (α = 2.6 ± 0.4), reducing ethanol content only slightly more than the bare PAN support itself (PAN exhibits α = 2).^16^ The addition of only 2 mol % NBF8 to the monomer and 7 mol % NBF8 to the polymer to achieve a fluorocarbon-dominated surface drastically reduces flux by ∼20× and increases α by ∼10×, demonstrating the effect of having a fluorocarbon-rich outer surface on a predominantly pNB bulk. Increasing fluorination results in a decrease in flux and an increase in selectivity until 63 mol % NBF8 in the polymer, beyond which increasing fluorination does not appear to statistically impact membrane performance. These membranes remain stable during testing in ethanol–water feed solutions, and ATR-IR scans of pNB, the 7% NBF8 copolymer, and pNB synthesized on PAN do not show compositional changes after 7 days when stored in a 90/10 v/v% ethanol–water solution, as shown in Figure S11.

**Figure 9:**
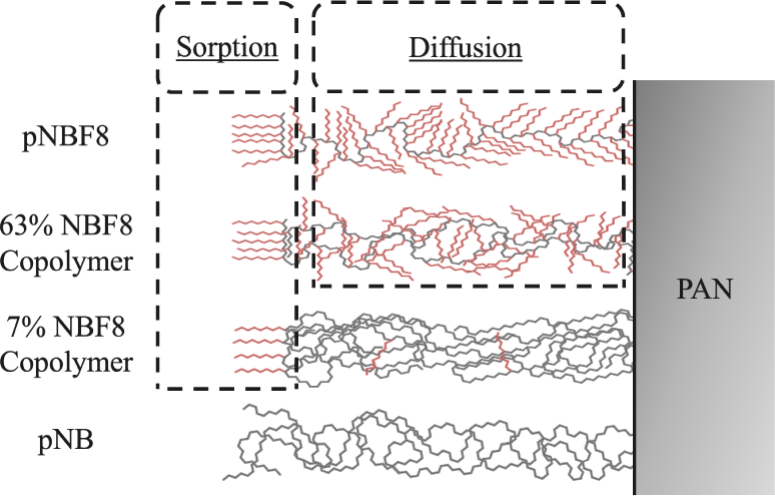
Schematic illustration of the relative concentration of perfluorooctane chains near the surface vs in the bulk for the 7% NBF8 copolymer, 63% NBF8 copolymer, and pNBF8 homopolymer. pNB is also shown for completeness. These surface and bulk regions are isolated by dashed boxes into the parts of the fluorinated membranes affecting sorption and diffusion. Hydrocarbon chains are shown in gray, and fluorocarbon chains are shown in red.

Figure 9 shows a model that we developed to explain the ethanol dehydration performance of p(NB-*co*-NBF8) membranes. In this model, the structural areas within the polymer membranes that are anticipated to affect sorption and diffusion are isolated for pNB, the 7% NBF8 copolymer, the 63% NBF8 copolymer, and pNBF8. For the 7% NBF8 copolymer, we assume that fluorination in the bulk is low enough that the diffusion of water and ethanol molecules will be similar to that in pNB. Therefore, the majority of the 7% NBF8 copolymer selectivity must come from the sorption of water over ethanol through the outer surface of the membrane. Based on water contact angle data in Figure 3a and ethanol contact angle data in Table S5, the outermost 0.5 nm of the 7% NBF8 copolymer membrane should be of similar structure to the 63% NBF8 copolymer and the pNBF8 homopolymer, making the additional selectivity for the 63% NBF8 copolymer and pNBF8 attributable to relative diffusion rates of water versus ethanol among fluorocarbon fractional free volumes.

To assess the individual sorption and diffusion components of pNB and pNBF8, a swelling experiment was performed to estimate the diffusion coefficients of water and ethanol in a pNB matrix. pNB films were placed in separate water and ethanol environments for 1 h to achieve the equilibrium sorption of the respective solvents. The films were then removed from the solvents, and desorption was measured as a function of time and polymer thickness, as shown in Figure S12. It has previously been shown that at short time periods after film removal from solvent, relative mass loss can be modeled by
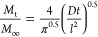
5where *M*_t_ is the mass desorbed from the film at time *t*, *M*_∞_ is the total mass desorbed from the film, *D* is the diffusion coefficient, *t* is the time after removal, and *l* is the polymer thickness.^69−71^ Estimates for *D* were obtained from kinetic curves of desorption in Figure S12 fitted to the above equation and are reported in the Section S11. Using the obtained diffusion values and the selectivity for pNB in pervaporation, a sorption ratio was easily calculated for pNB. To obtain a water-to-ethanol diffusion ratio for pNBF8, we assumed that the diffusion ratio for pNB is the same diffusion ratio as the 7% NBF8 copolymer, given that the 7% NBF8 copolymer is predominantly hydrocarbon outside of the surface region. Taking the diffusion ratio and the selectivity for the 7% NBF8 copolymer, the effect of having a fluorocarbon-dominated surface on sorption selectivity was calculated. To complete the study, the selectivity for a pNBF8 membrane was divided by the sorption ratio of water to ethanol for a fluorocarbon-dominated surface in the 7% NBF8 copolymer to obtain the diffusional selectivity for a pNBF8 membrane. The calculation pathway through Figure 9 is shown in Figure S13, and the diffusion and sorption ratios of water to ethanol for pNB and pNBF8 are shown in Table 2.

**Table 2:** Sorption and Diffusion Component Selectivity Estimates for pNB and pNBF8

Film	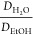	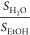
pNB	1.6 ± 0.4	1.6 ± 0.5
pNBF8	9 ± 6	18 ± 6

As expected, the sorption and diffusion components are more selective for pNBF8 than pNB, implying that both the sorption and diffusion properties of pNBF8 are necessary to most selectively permeate water over ethanol in this copolymer system. Approximately equivalent values for selectivity and percent ethanol content in the permeate for the 63% NBF8 copolymer to those of the pNBF8 homopolymer, however, suggest that full fluorination is not required to obtain these values. Rather, a fully fluorinated surface, which can be achieved with as low as 7 mol % NBF8 in the copolymer (or 2 mol % NBF8 in the monomer), is necessary to obtain the sorption selectivity, and 63 mol % NBF8 in the copolymer (or 50 mol % in the monomer) generates a bulk copolymer film that is similar enough in structure to mimic the diffusional properties of water and ethanol through pNBF8.

## Conclusions

Conventional methods for the synthesis of fluorocarbon thin films and membranes are time-intensive, require excessive amounts of solvent, and often use significant amounts of environmentally hazardous PFAS. The scROMP approach was implemented as a method to significantly reduce the amount of time, solvent, and PFAS necessary to synthesize low surface energy polymer films. Copolymerization of 5-(perfluoro-*n*-alkyl)norbornenes (*n* = 4, 6, and 8) with norbornene using scROMP allows for the combination of polymer film synthesis and deposition in <2 min with <1 mL of solvent. The fluorinated component tends to dominate the surface, with *n* = 4, 6, and 8 maintaining water and hexadecane contact angles comparable to their respective homopolymers with ≤10 mol % fluorocarbon incorporation in the bulk film and as low as 2 mol % fluorocarbon monomer in the comonomer solution. Contact angles with hexadecane showed that the p(NB-*co*-NBF*n*) copolymers dilute in fluorocarbon are stimuli-responsive and will switch surface groups to more omniphilic hydrocarbon groups when probed by a hydrocarbon liquid. XPS and SEM-EDS further demonstrated the presence of a fluorocarbon-rich region near the surface of the 7% NBF8 copolymer.

p(NB-*co*-NBF*n*) copolymer thin films successfully performed as membranes for ethanol dehydration, achieving selectivities as high as those of the semifluorinated homopolymer while using significantly less PFAS in their synthesis. The strong fluorocarbon surface segregation coupled with dilute bulk fluorocarbon of the 7% NBF8 copolymer provided a framework to estimate the sorption and diffusion components of permeability for pNB and pNBF8, showing that a fluorocarbon outer layer increases selectivity by ∼10×, and increasing fluorination to 63 mol % NBF8 in the bulk increases selectivity by another ∼5× relative to those for pNB. These results provide quantitative evidence for the surface and bulk roles of fluorocarbon chains in ethanol dehydration and, with that, new insight into explaining the surprising utility of semifluorinated systems and the more commonly studied fully fluorinated ones to selectively pass water over a more wettable polar liquid. From a sustainability standpoint, the scROMP method enables remarkably rapid synthesis of copolymer films and membranes to facilitate evaluation and materials discovery while negating the need for a corresponding number of large-scale syntheses with their high usage of materials, solvent, and time. The specific approach here achieves twenty-one different copolymer films to pinpoint optimal levels of fluorination while using significantly less solvent and fluorocarbon than traditional syntheses of perfluoropolymer selective layers.

## Supporting Information Available

^1^H NMR data and calculations, a Zisman plot, an Owens–Wendt plot, a contact angle stability study, PFAS usage comparison calculations, selected DSC data, an XPS survey spectrum for pNB, calculations of the mean free path of electrons for XPS, a SEM-EDS spectrum, an ethanol dehydration fatigue test, ethanol contact angles, a diffusion experiment for pNB, and pNB and pNBF8 sorption and diffusion ratio calculations (PDF)
