# Supplementary material for: Fluorocarbon Minimization Via Semifluorinated Copolymer Films by Combining Spin Coating and Ring-Opening Metathesis Polymerization
Source: Langmuir. 2025 Mar 5;41(10):6931–43. doi: 10.1021/acs.langmuir.4c05253 (PMC11924226; doi:10.1021/acs.langmuir.4c05253)
Supplement: Supplementary file 1 — la4c05253_si_001.pdf [file la4c05253_si_001.pdf]

# Supporting Information

# Fluorocarbon Minimization via Semifluorinated Copolymer Films by Combining Spin Coating and Ring-Opening Metathesis Polymerization

*Matthew P. Vasuta,[1] Zane J. Parkerson,[2] Tyler D. Oddo,[2] Bridget R. Rogers,[2] and G. Kane Jennings[2,*]*

[1]Interdisciplinary Materials Science Program, Vanderbilt University, Nashville, TN, 37235

[2]Department of Chemical and Biomolecular Engineering, Vanderbilt University, Nashville, TN 37235

**Corresponding Author**: *G. Kane Jennings, Department of Chemical and Biomolecular Engineering, Vanderbilt University, Nashville, TN, 37235; Email: kane.g.jennings@vanderbilt.edu

**S1: Mol % NBF*n* from $^1$H NMR**

$^1$H NMR was used to obtain compositional data of the p(NB-co-NBF*n*) films. The $^1$H NMR spectrum of pNB shown in Figure S1 is consistent with previous literature:[1] ($CDCl_3$) δ *cis*: 5.21 (2H), 2.78 (2H), 1.83 (3H), 1.35 (2H), 1.03 (1H); *trans*: 5.34 (2H), 2.43 (2H), 1.83 (3H), 1.35 (2H), 1.03 (1H). Moisture from $CDCl_3$ appears at 1.55 ppm, and pentane absorbed in the film from dissolving the monomers appears at 1.30, 1.26, and 0.88 ppm.

Figure S1 also displays the $^1$H spectra of pNBF4 and p(NB-co-NBF4) at monomer molar ratios of 1:3 to 100:1 NB:NBF4. Figure S2 displays the $^1$H NMR spectra for p(NB-co-NBF6) with the pNB and pNBF6 homopolymers, and Figure S3 displays the $^1$H NMR spectra for p(NB-co-NBF8) with the pNB and pNBF8 homopolymers. Solvent peaks are capped to not interfere with other spectra.

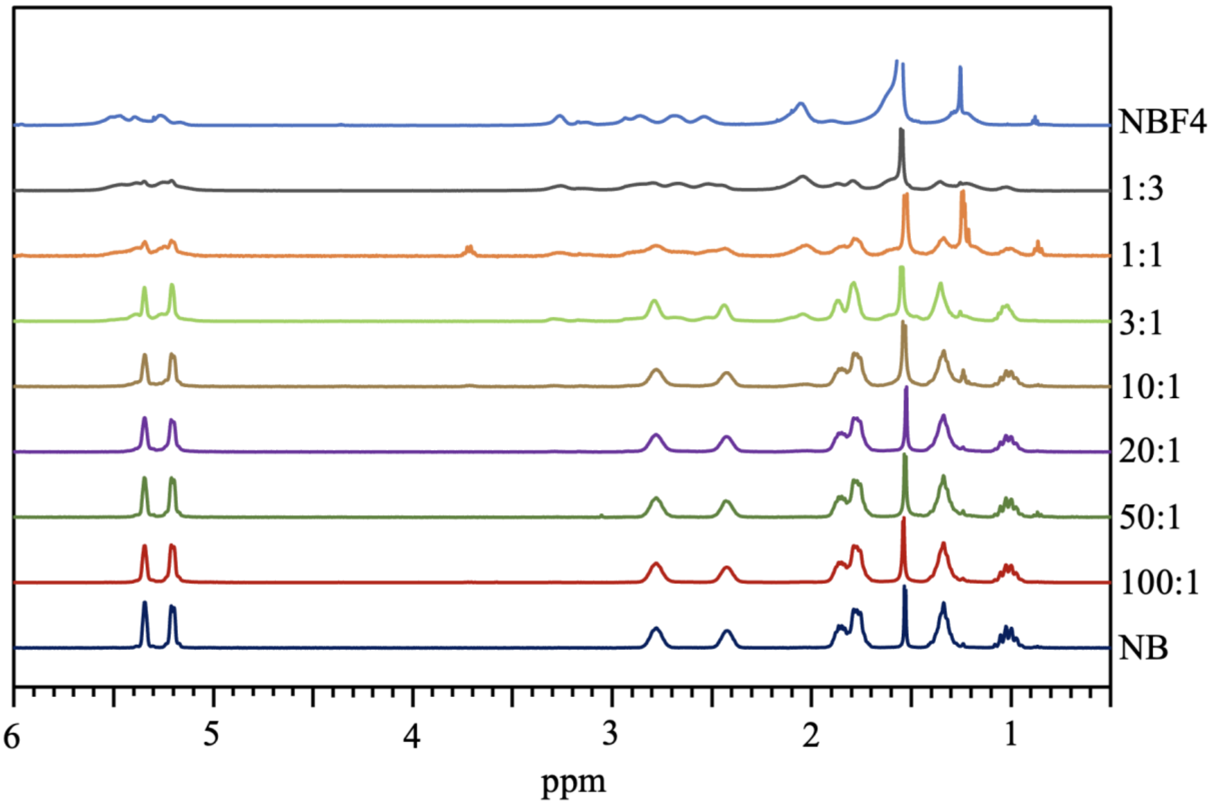


**Figure S1.** $^1$H NMR of p(NB-co-NBF4) with pNB and pNBF4 homopolymers. Monomer molar ratios are listed as NB:NBF4. All polymer films were dissolved in $CDCl_3$. Solvent peaks are capped to better visualize the spectra together. The areas for the capped peaks at 1.56 ppm are 0.66 (NB), 1.58 (100:1), 0.58 (50:1), 0.48 (20:1), 1.72 (10:1), 1.68 (3:1), 1.68 (1:1), 1.34 (1:3), and 4.27 (NBF4) relative to the area of the olefinic region between 5 and 6 ppm.

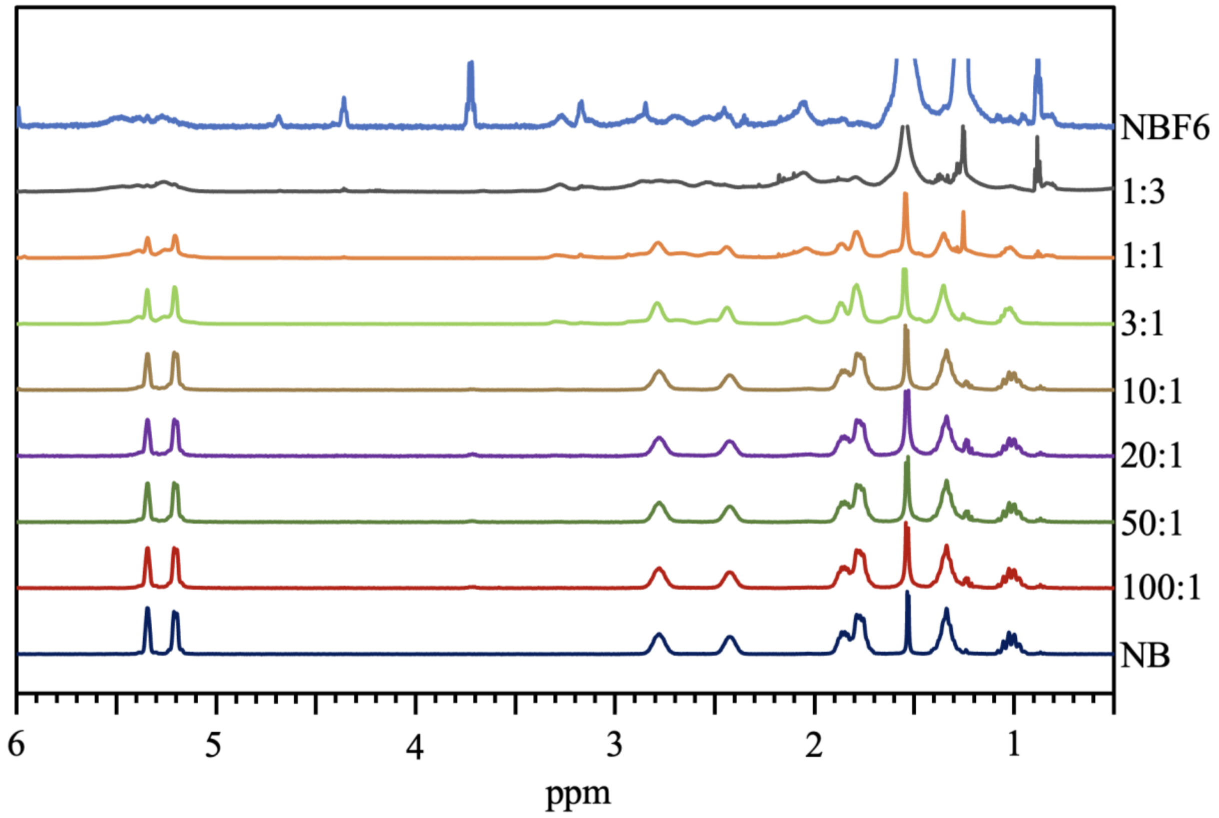


**Figure S2.** $^{1}$H NMR of p(NB-co-NBF6) with pNB and pNBF6 homopolymers. Monomer molar ratios are listed as NB:NBF6. All polymer films were dissolved in $CDCl_3$. Solvent peaks are capped to better visualize the spectra together. The areas for the capped peaks at 1.56 ppm are 0.66 (NB), 0.92 (100:1), 1.56 (50:1), 2.53 (20:1), 1.01 (10:1), 2.59 (3:1), 0.65 (1:1), 4.94 (1:3), and 17.54 (NBF4) relative to the area of the olefinic region between 5 and 6 ppm. The area of the 2$^{nd}$ capped peak in the NBF6 spectra is 10.42 relative to the same olefinic region.

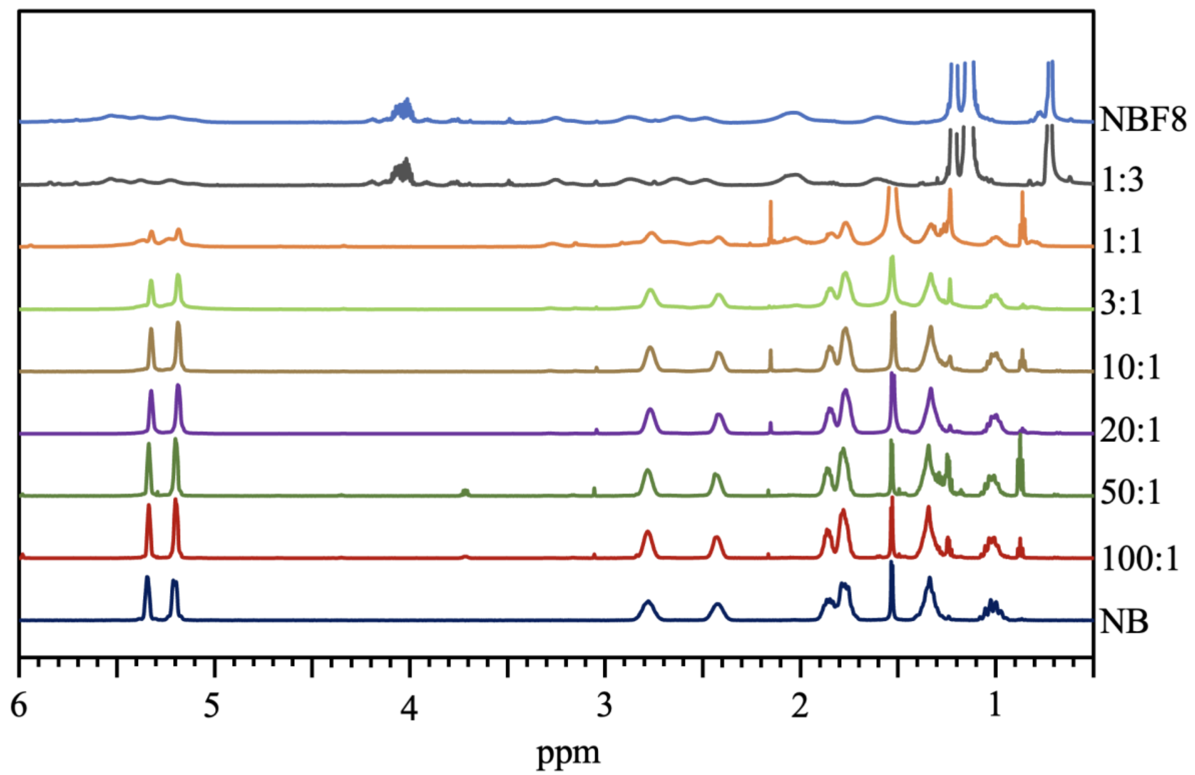


**Figure S3.** $^1$H NMR of p(NB-co-NBF8) with pNB and pNBF8 homopolymers. Monomer molar ratios are listed as NB:NBF8. All polymer films were dissolved in $CDCl_3$ except for 1:3 NB:NBF8 and pNBF8, which were dissolved in 600 µL perfluoro(methylcyclohexane) with a 100 µL hexane-d14 spike. Solvent peaks are capped to better visualize the spectra together. The areas for the capped peaks at 1.56 ppm are 0.66 (NB), 0.94 (100:1), 1.31 (50:1), 1.65 (20:1), 1.70 (10:1), 0.98 (3:1), and 3.12 (1:1) relative to the area of the olefinic region between 5 and 6 ppm. The areas for the capped peaks at 1.3 ppm and 0.5 ppm are 5.26 and 3.08 (1:3) and 4.72 and 2.18 (NBF8) relative to the same olefinic region.

Due to the extremely low solubility of p(NB-co-NBF8) films of high NBF8 content in almost all deuterated solvents, a mixture of perfluoro(methylcyclohexane) and deuterated hexane was used to dissolve films synthesized from 1:3 NB:NBF8 and NBF8 monomer solutions to generate their respective spectra. A few additional solvent peaks appear in the 1:3 NB:NBF8 and NBF8 spectra, so Figure S4 shows $^1$H NMR spectra obtained from 1) only d-hexane and 2) perfluoro(methylcyclohexane) with a d-hexane spike to investigate solvent interference.

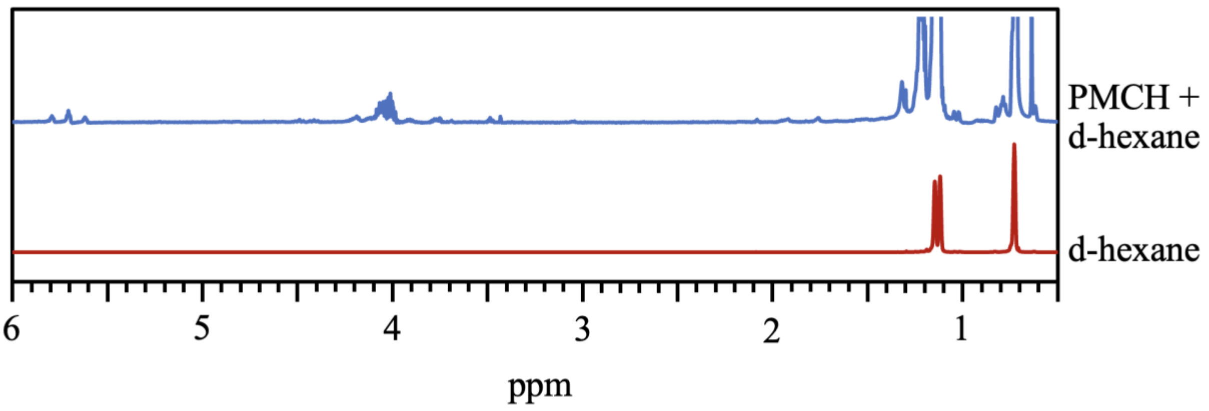


**Figure S4.** $^1$H NMR of the solvents used with the 1:3 NB:NBF8 and pure NBF8 monomer compositions. PMCH + d-hexane sampled 600 µL of perfluoromethylcyclohexane and 100 µL of deuterated hexane. d-Hexane sampled 700 µL of deuterated hexane. The areas for the capped peaks at 1.3 ppm and 0.5 ppm in the PMCH + d-hexane spectrum are 39.71 and 19.78 relative to the area of the impurity at 4.05 ppm.

The peaks at 1.13, 1.11, and 0.73 ppm in the d-hexane spectrum in Figure S4 are attributed to non-deuterated hexane formed by hydrogen-deuterium exchange from trace water in the hygroscopic d-hexane. The multiplet at 4.1 ppm does not appear in the d-hexane spectrum and is instead only observed in the combined perfluoro(methylcyclohexane) and d-hexane spectrum. A chemical shift in this region is consistent with a C-F bond at the α-position relative to a methyl or methylene group, implying that a small amount of hydrogen-fluorine exchange[2–4] occurs between the perfluoro(methylcyclohexane) and d-hexane. Fortunately, perfluoro(methylcyclohexane) and d-hexane interference is distinct from any regions used for integration and is not expected to influence any reported numerical values.

Our previous study[5] included a heteronuclear single quantum coherence (HSQC) experiment and peak assignments for several of the protons in pNBF4. *Cis*/*trans* isomerization and varied placement of the perfluoro chain shift and broaden proton peaks, complicating the p(NB-co-NBF*n*) spectra. Therefore, a range of values in Figure S5 is assigned for each proton in a NBF*n*

repeat unit: 5.7-5.1 ppm ($H_a$, $H_b$), 3.4-3.0 ppm ($H_i$), 3.0-2.4 ppm ($H_c$, $H_d$), 2.2-1.9 ppm ($H_e$, $H_g$), 1.7-1.5 ppm ($H_h$), and 1.3-1.1 ($H_f$).

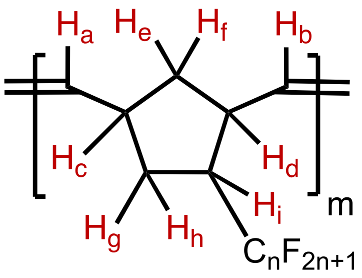


**Figure S5.** Nonequivalent protons on a NBF*n* repeat unit. Note that *cis*/*trans* isomerization and varied placement of the perfluoro chain shift and broaden proton peaks.

Since the pNBF*n* spectra do not show strong signals between 1.9 and 1.7 ppm, mol % NBF*n* in this work was determined by comparing the peak area ratio of the aliphatic protons of pNB between 1.9 and 1.7 ppm and the olefinic region between 5.5 and 5.0 ppm that includes both pNB and pNBF*n* components.

$$Mol\ \%\ NBFn = 100\% - \frac{\frac{A_{1.9\ to\ 1.7\ ppm\ p(NB-co-NBFn)}}{A_{5.5\ to\ 5.0\ ppm\ p(NB-co-NBFn)}}}{\frac{A_{1.9\ to\ 1.7\ ppm\ pNB}}{A_{5.5\ to\ 5.0\ ppm\ pNB}}} * 100\% \quad (S1)$$

The ratio for the pNB spectrum was assumed to be 0 mol % NBF*n*, and the ratios of the homopolymer pNBF*n* spectra were assumed to be 100 mol % NBF*n* for each system, with mol % NBF*n* scaling linearly with peak area ratios. Mol % NBF*n* for different *n* at different monomer molar ratios are displayed in Table S1. For all chain lengths and monomer ratios, preferential incorporation of the NBF*n* repeat unit over the NB repeat unit is observed.

**Table S1.** Mol % NBF*n* in the bulk polymer for various monomer ratios of p(NB-co-NBF4), p(NB-co-NBF6), and p(NB-co-NBF8) determined from $^1$H NMR.

| NB:NBF*n* Ratio (% NBF*n*) in the Monomer Solution | Mol % NBF*n* | | |
|---|---|---|---|
| | p(NB-co-NBF4) | p(NB-co-NBF6) | p(NB-co-NBF8) |
| 1:3 (75) | 97 | 91 | 95 |
| 1:1 (50) | 57 | 58 | 62 |
| 3:1 (25) | 47 | 34 | 38 |
| 10:1 (9) | 10 | 14 | 14 |
| 20:1 (5) | 8 | 10 | 10 |
| 50:1 (2) | 6 | 7 | 7 |
| 100:1 (1) | 4 | 4 | 4 |

To investigate how incorporation evolves with polymerization time, Table S2 shows how mol % NBF8 changes with polymerization time on the 50:1 NB:NBF8 film terminated at different times using ethyl vinyl ether.

**Table S2.** Mol % NBF8 for films with initial molar concentration of 50:1 NB:NBF8 terminated by spin coating 1 mL of ethyl vinyl ether after polymerization times of 10, 30, 60, and 90 s.

| Polymerization Time (s) | Mol % NBF8 |
|---|---|
| 10 | 1.5 |
| 30 | 2.8 |
| 60 | 3.4 |
| 90 | 3.3 |

Mol % NBF8 of films synthesized from the 50:1 NB:NBF8 solution increases with polymerization time, suggesting that the NBF8 monomer becomes better entrained in the growing film with polymerization time. This explanation is further supported by the increase of mol % NBF8 in the polymer film to 7% when the 50:1 NB:NBF8 film is spun for 60 s without ethyl vinyl ether termination as shown in Table S1. This film would have the longest reaction time, and therefore

the greatest ability to polymerize NBF8 monomers that may be driven towards the surface of the film.

**S2: Surface Energies of pNB and pNBF*n* Films**

To determine the critical surface tensions of pNBF*n* homopolymer films, the Zisman method was employed.[6] Advancing contact angles using a sequence of *n*-alkane probe liquids (*n* = 6, 8, 10, 12, 14, & 16) were measured on pNBF4, pNBF6, and pNBF8 homopolymer films, and the cosines of their values are plotted in Figure S6.

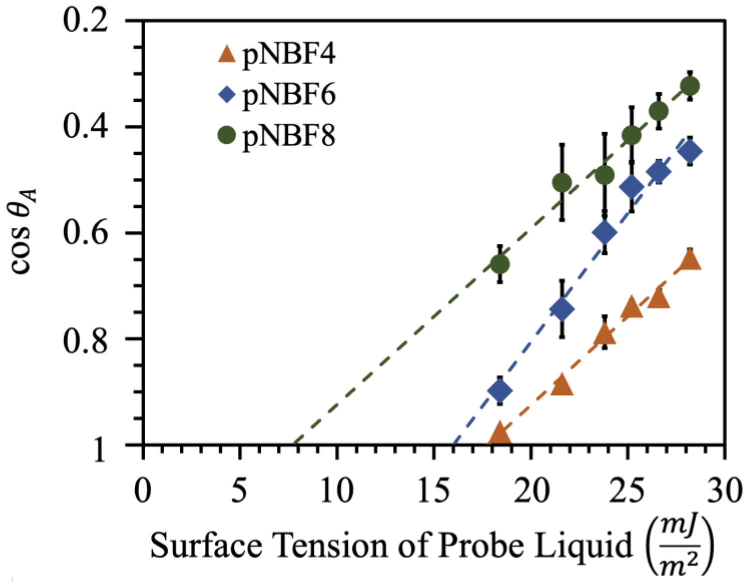


**Figure S6.** Zisman plot for pNBF4, pNBF6, and pNBF8 homopolymers. Probe liquids were a series of *n*-alkanes (*n* = 6, 8, 10, 12, 14, & 16). The x-intercepts of the lines of best fit were used to determine the critical surface tension ($\gamma_c$) for pNBF4, pNBF6, and pNBF8. If an error bar is not visible, the error is represented by the size of the symbol.

Per the Zisman method, a linear extrapolation of the line of best fit to the point where $\cos\theta_A = 1$ gives the critical surface tension ($\gamma_c$) where any liquid with surface tension below the set $\gamma_c$ will completely wet the surface. For a system with only dispersive components, critical surface tension is approximately equivalent to the surface energy of the solid. For the pNBF$n$ homopolymers,

critical surface tensions, and therefore surface energies, were 18, 16, and 8 $\frac{mJ}{m^2}$ for pNBF4, pNBF6, and pNBF8, consistent with fluorocarbon-dominated surfaces.

Since the probe liquids used in the Zisman plot all completely wet the surface of pNB, the Owens-Wendt method[7] was instead used to calculate the surface energy of pNB. Per the Owens-Wendt method, contact angles for a series of liquids with different ratios of dispersive to polar surface tensions ($\gamma_{LV}^{D}$:$\gamma_{LV}^{P}$) were taken along the surface of the polymer film. Assuming that the solid-vapor interfacial energy ($\gamma_{SV}$) is the sum of the dispersive and polar solid-vapor interfacial energies ($\gamma_{SV}^{D}$ and $\gamma_{SV}^{P}$), and $\theta$ is the angle of the three-point contact line between solid, liquid, and vapor, when $\frac{\gamma_{LV}(1+\cos\theta)}{2\sqrt{\gamma_{LV}^{D}}}$ is plotted against $\sqrt{\frac{\gamma_{LV}^{P}}{\gamma_{LV}^{D}}}$ as is seen in Figure S7 for pNB, the y-intercept of the line of best fit should be $\sim\sqrt{\gamma_{SV}^{D}}$ for the polymer film. Since pNB contains only dispersive components, $\gamma_{SV}^{D} = \gamma_{S} \sim 37\,\frac{mJ}{m^2}$.

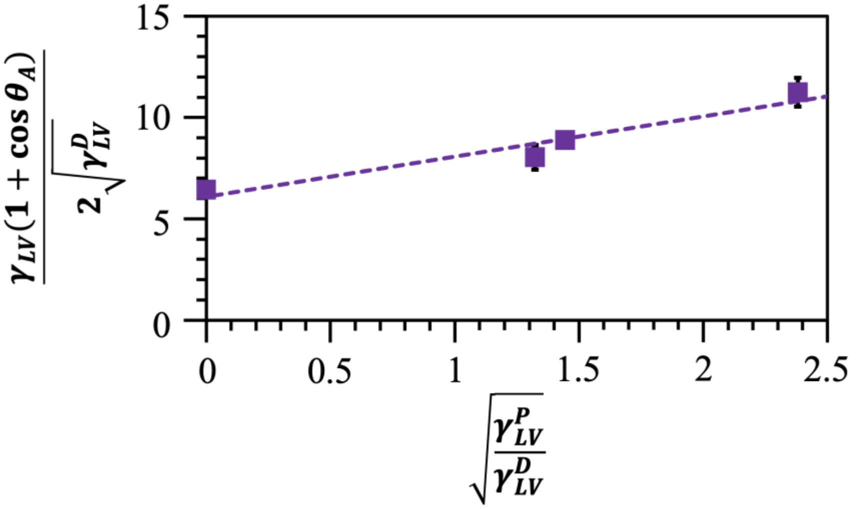


**Figure S7.** Owens-Wendt plot for pNB homopolymer. Probe liquids were water, diiodomethane, glycerol, and ethylene glycol. The y-intercept of the line of best fit was used to calculate $\gamma_s$ for pNB. If an error bar is not visible, the error is represented by the size of the symbol.

**S3: Contact Angle Stability with Extended Exposure Time**

To verify the stability of p(NB-co-NBF$n$) films to maintain low surface energies after long time periods, contact angles were measured after 7 days in a closed vial for the 7% NBF8 copolymer and pNBF8 films and are shown in Table S3.

**Table S3.** Water and hexadecane contact angles for the 7% NBF8 copolymer and pNBF8 films within 2 h of synthesis (New) and after 7 days in a closed vial.

| Film | $\theta_A$ $H_2O$ (°) | $\theta_A$ HD (°) |
|---|---|---|
| 7% NBF8 Copolymer, New | 121 ± 2 | 71 ± 3 |
| 7% NBF8 Copolymer, After 7 days | 118 ± 4 | 70 ± 2 |
| pNBF8, New | 118 ± 1 | 71 ± 2 |
| pNBF8, After 7 days | 119 ± 3 | 70 ± 2 |

**S4: Comparison of PFAS Usage in the Synthesis of p(NB-co-NBF$n$) Films using scROMP versus Traditional Methods**

We estimated the number of moles of PFAS and volume of PFAS-enriched waste to synthesize a 2 cm x 2 cm polymer film by this work and by the surface-initiated atom transfer radical polymerization and postpolymerization modifications reported by Brantley et al.[8] In their work, the polymer films were acylated by immersion in a pentafluorobenzoyl chloride solution with dichloromethane as the solvent:

$$(20\ mM\ pentafluorobenzoyl\ chloride)(5\ mL\ DCM) = 1\ x\ 10^{-4}\ mol\ PFAS \quad (S2)$$

To form the end copolymer with wetting properties similar to the 7% NBF8 copolymer film in this study, Brantley et al.'s films were again immersed in a perfluorobenzoyl chloride solution of slightly elevated concentration:

$$(25\ mM\ pentafluorobenzoyl\ chloride)(5\ mL\ DCM) = 1.3\ x\ 10^{-4}\ mol\ PFAS \quad (S3)$$

For the 7% NBF8 copolymer synthesized by scROMP, the moles of PFAS are determined by multiplying the volume of NBF8 dispensed by the neat NBF8 concentration.

$$(4\ \mu L\ NBF8)\left(\frac{2.7\ mol\ NBF8}{L\ NBF8}\right) = 1.1\ x\ 10^{-5}\ mol\ PFAS \quad (S4)$$

The estimated total moles of PFAS and total volume of PFAS-enriched waste are displayed in Table S4 along with the usage amounts for the synthesis of the 7% NBF8 copolymer in the present study.

**Table S4.** Usage of PFAS in the synthesis of semifluorinated films with -$CF_3$ dominated surfaces.

| Method | Moles of PFAS Used | Volume of PFAS-Enriched Waste (mL) |
|---|---|---|
| Brantley et al. | 2.3 x $10^{-4}$ | 10 |
| This Work | 1.1 x $10^{-5}$ | 0.2 |

An estimated ~20x decrease in moles of PFAS used and ~50x decrease in volume of PFAS-enriched waste are achieved by using scROMP to generated a fluorocarbon surface over traditional methods.

**S5: Differential Scanning Calorimetry of the 1) pNBF8 Homopolymer and 2) Copolymers with Fluorocarbon-Dominated Surfaces but Low Bulk Fluorination**

To investigate the effects of copolymerization on polymer phase, pNBF8 and films with the lowest level of bulk fluorination to achieve fluorocarbon-dominated surfaces for each of the p(NB-co-NBF*n*) films were tested using DSC and are shown in Figure S8.

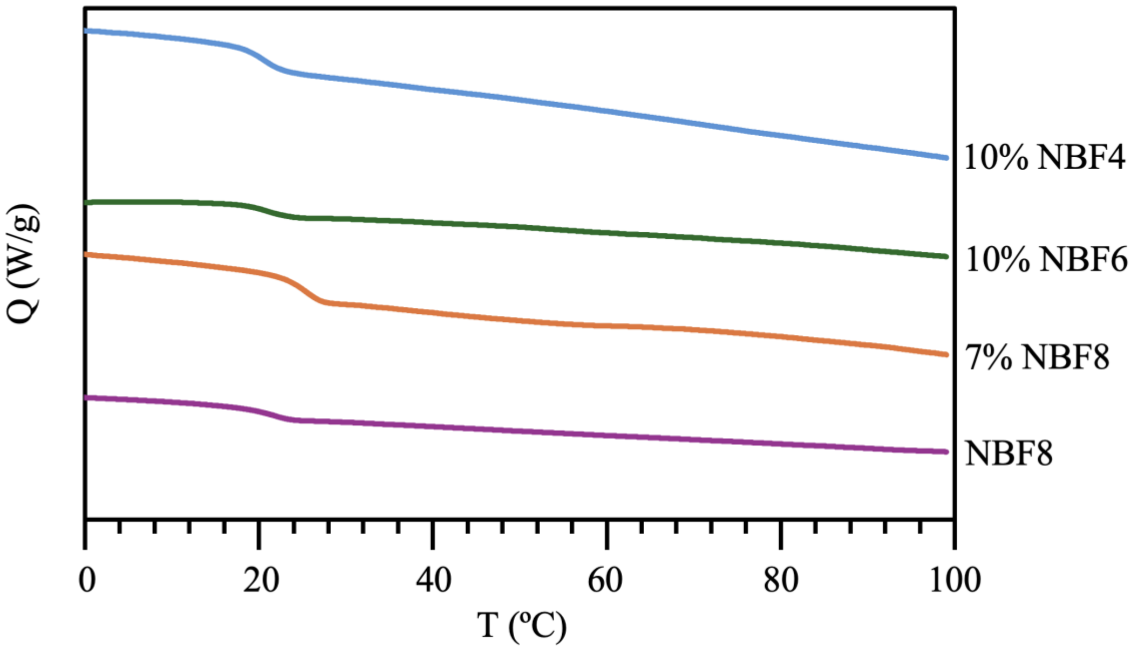


**Figure S8.** DSC curves for the 10% NBF4, 10% NBF6, and 7% NBF8 copolymers along with the pNBF8 homopolymer. Displayed curves represent the second heating cycle after erasing thermal history.

Inflection points representing the glass transition temperatures of the polymers are 42°C, 43°C, 51°C, and 48°C for the 7% NBF4 copolymer, 10% NBF6 copolymer, 7% NBF8 copolymer, and pNBF8 homopolymer, respectively, indicating that p(NB-co-NBF*n*) films are in the glassy state for characterization techniques at room temperature, but all p(NB-co-NBF8) films are in the rubbery state when undergoing ethanol dehydration at 60°C.

**S6: Survey XPS Spectrum for pNB**

A XPS survey spectrum was taken for pNB and is shown in Figure S9. The C 1s peak is visible at 286 eV, and the O 1s peak appears at 531 eV. Oxygen may appear due to $SiO_2$ formation, oxidation of some of the olefinic bonds in the polymer backbone,[9] or adventitious hydrocarbons adsorbed on the surface due to atmospheric exposure.

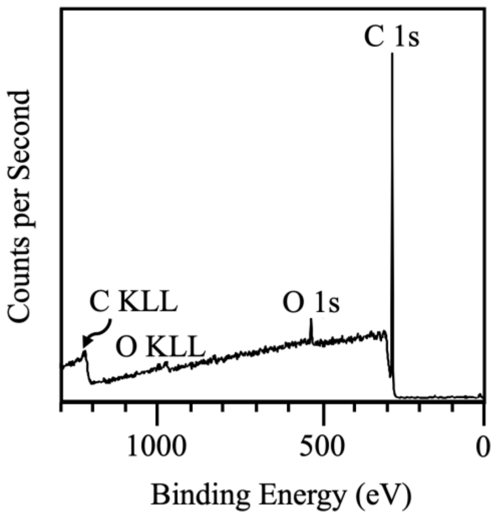


**Figure S9.** XPS spectra for pNB at a 45° take-off angle.

**S7: Mean Free Paths for Fluorocarbon-Rich Layer Depth Estimation from XPS**

Mean free paths of electrons were estimated from Seah et al.'s standard data base for mean free path vs. kinetic energy of electrons.[10] The carbon 1s photoelectron peak appears at a binding energy of ~284 eV, and the kinetic energy of C 1s electrons can be determined as follows:

$$Kinetic\ Energy\ of\ Electron = Photon\ Energy - Binding\ Energy \quad (S5)$$

$$Kinetic\ Energy\ of\ Electron = 1486\ eV - 284\ eV$$

$$Kinetic\ Energy\ of\ Electron = 1202\ eV$$

From Seah et al., 1202 eV corresponds to a mean free electron path of ~1.5 nm. XPS signal intensity originates within approximately three mean free paths from the surface.[11] Therefore, when the take-off angle is 90°, information from the top ~4.5 nm is acquired. Information is acquired from the top ~3.2 and 2.3 nm when the take-off angle is 45° and 30°, respectively.

**S8: SEM-EDS Spectrum for 7% NBF8 copolymer**

Figure S10 displays the EDS spectrum for the EDS maps of the 7% NBF8 copolymer shown in Figure 7. As was demonstrated with other techniques, the films are predominately carbon

with smaller amounts of oxygen and fluorine. Gold and silicon also appear in the spectra from the Au-coated silicon substrate.

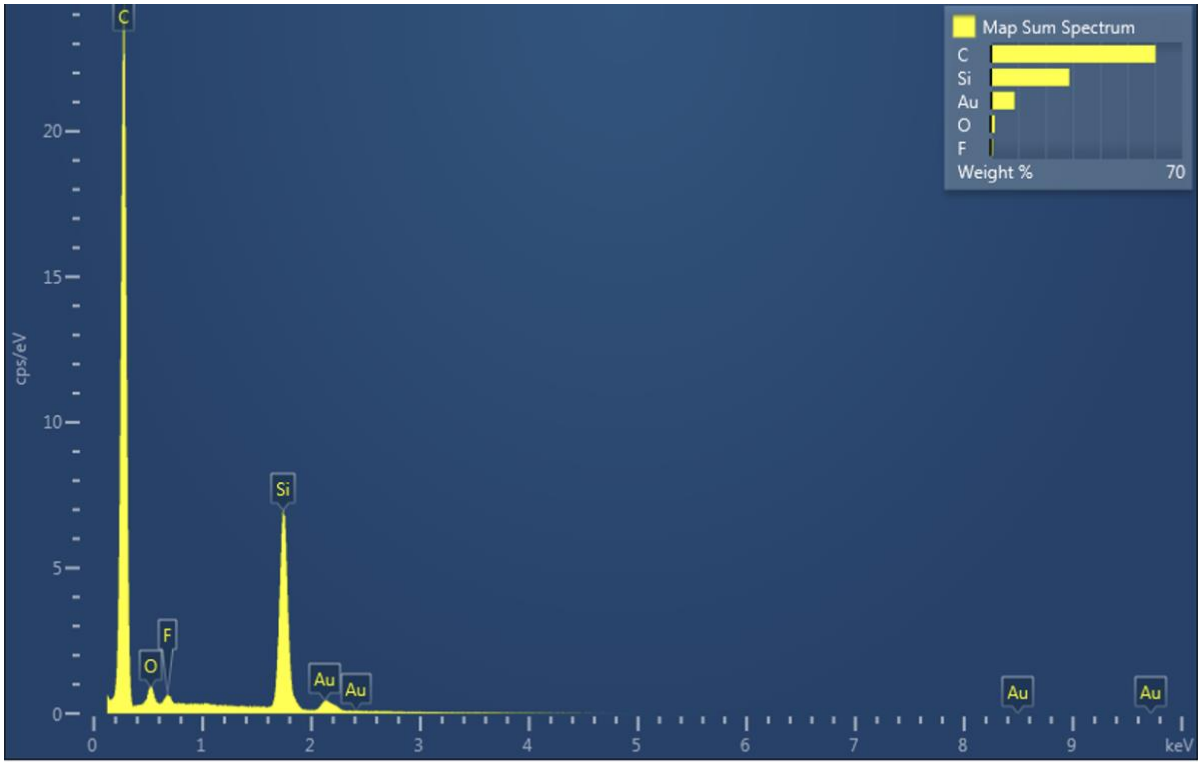


**Figure S10.** EDS spectra for 7% NBF8 copolymer images in Figure 7. Carbon, gold, and fluorine elemental maps are shown in Figure 7, while silicon appears in the spectra because the films were synthesized atop gold-coated silicon wafers, and oxygen appears either from $SiO_2$, oxidation of some of the olefinic backbones, or adventitious hydrocarbons.

**S9: Ethanol Dehydration Fatigue Test**

To ensure that the p(NB-co-NBF*n*) films did not degrade in ethanol dehydration, ATR-IR scans of the pNB, 7% NBF8 copolymer, and pNBF8 were obtained to ensure no loss of hydrocarbon or fluorocarbon parts and are shown in Figure S11.

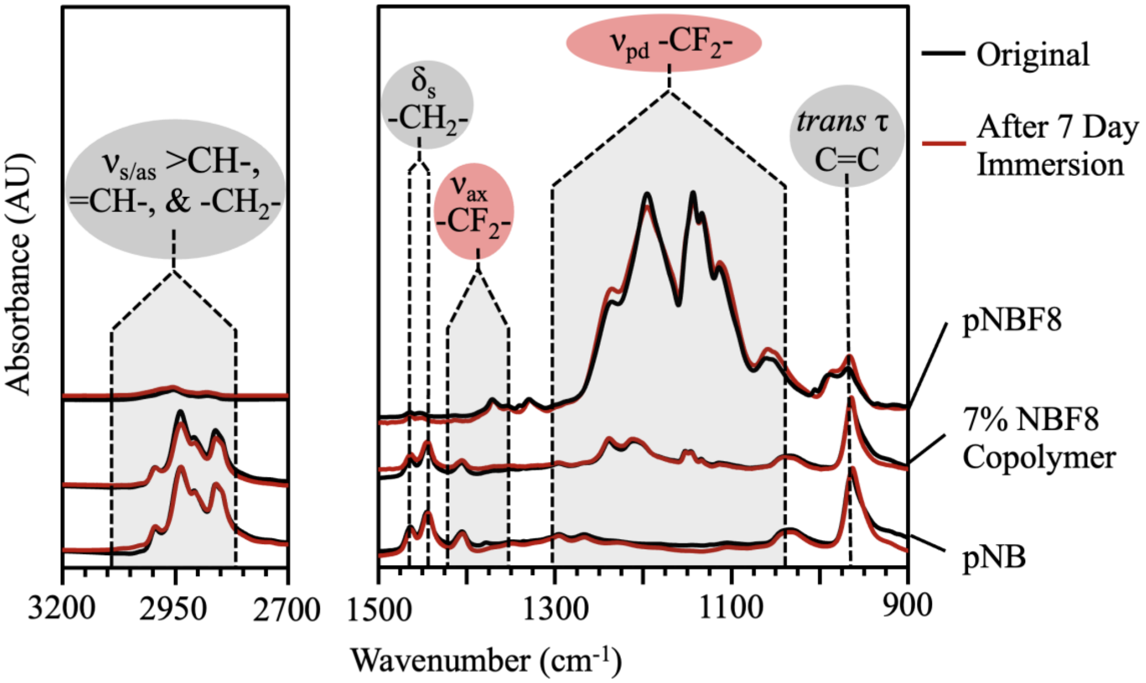


**Figure S11.** ATR-IR spectra of the pNB, 7% NBF8 copolymer, and pNBF8 films after 7 days storage in 90/10 v/v % ethanol to water.

**S10: Ethanol Contact Angles**

For the membrane model in Figure 9, the surfaces of the 7% NBF8 copolymer and 63% NBF8 copolymer were assumed to produce the same sorption effect as the pNBF8 homopolymer when in contact with a 90/10 mass ratio of ethanol to water. Water contact angles in Figure 3a do not deviate between the two polymers, and ethanol contact angles are shown in Table S5 to demonstrate that the 7% NBF8 and 63% NBF8 copolymer surface show the same affinity for ethanol as the pNBF8 homopolymer. These results are in contrast to the data in Figure 4 for hexadecane, which show behavioral differences in advancing contact angles between the pNBF8 and 7% NBF8 copolymer.

**Table S5.** Advancing ethanol contact angles for the 7% NBF8 copolymer, 63% NBF8 copolymer, and pNBF8 obtained to ensure that molecular reorientation did not occur with ethanol as a probe liquid as was observed with hydrocarbon probe liquids.

| Film | $\theta_A$ EtOH (°) |
|---|---|
| 7% NBF8 Copolymer | 58 ± 3 |
| 63% NBF8 Copolymer | 59 ± 3 |
| pNBF8 | 61 ± 1 |

## S11: pNB and pNBF8 $\frac{D_{H_2O}}{D_{EtOH}}$ and $\frac{S_{H_2O}}{S_{EtOH}}$ Calculations

A common method to obtain diffusion coefficient estimates in polymer films is to track weight loss during solvent evaporation through kinetic curves of desorption.[12-14] Fick's 2nd law can be manipulated to yield an expression for relative weight loss $\left(\frac{M_t}{M_\infty}\right)$:

$$\frac{M_t}{M_\infty} = 1 - \frac{8}{\pi^2}\sum_{n=0}^{\infty}\frac{1}{(2n+1)^2}\exp\left[\frac{-D(2n+1)^2\pi^2 t}{l^2}\right] \quad (S6)$$

where $M_t$ is the mass desorbed from the film at time $t$, and $M_\infty$ is the total mass desorbed from the film, $D$ is the diffusion coefficient for water or ethanol, $l$ is the thickness of the polymer film, and $t$ is the time after removal.[14] For most polymer systems at short time periods, Fickian behavior is observed such that $\frac{M_t}{M_\infty}$ becomes approximately linear with $\frac{t^{0.5}}{l}$ and the above equation reduces to:[14]

$$\frac{M_t}{M_\infty} = \frac{4}{\pi^{0.5}}\left(\frac{Dt}{l^2}\right)^{0.5} \quad (S7)$$

By plotting $\frac{M_t}{M_\infty}$ against $\frac{t^{0.5}}{l}$ for a film removed from solvent, the diffusion coefficient can be estimated through the slope of the line of best fit for the linear portion of the graph:

$$D = \frac{\pi}{16}m^2 \quad (S8)$$

where $m$ is the slope of the line of best fit when the graph is linear.[12-14] To obtain diffusion coefficient estimates for our p(NB-co-NBF8) systems, pNB was swelled in water and ethanol environments and the kinetic curves of desorption are shown in Figure S11.

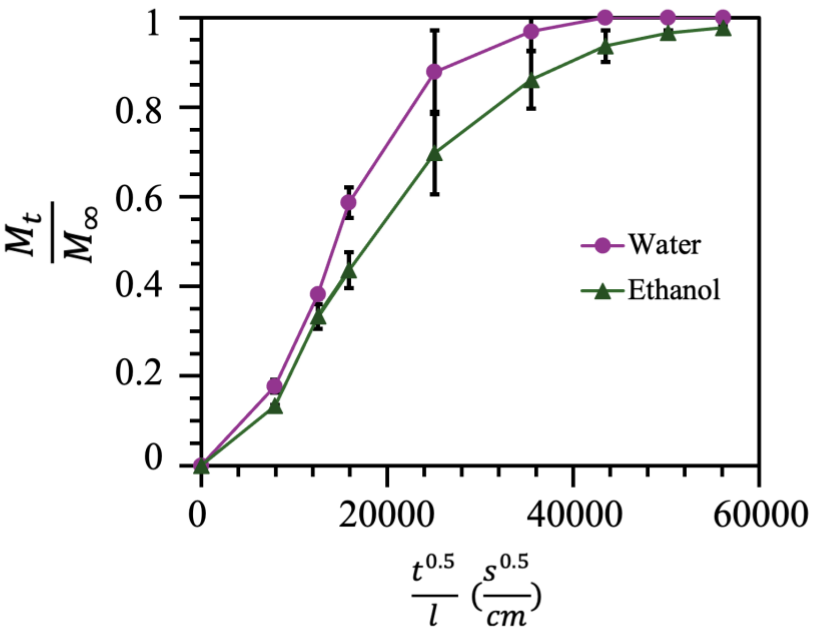


**Figure S12.** Kinetic curves of desorption for water and ethanol in a pNB film used to estimate diffusion coefficients. The thickness used for the x-axis, $l$, was taken from profilometry on polymer films synthesized on 6 cm x 6 cm Si substrates using 400 µL dispenses at 1500 RPM spin speed and was determined to be 7.2 µm. If an error bar is not visible, the error is represented by the size of the symbol.

The first four points for both water and ethanol were assumed to be the linear portion of the graph displaying Fickian behavior, so diffusion estimates were taken from these points. Slope values were taken from the least-squares line of best fit through the origin for these points, giving (2.3 ± 0.4) x $10^{-10} \frac{cm^2}{s}$ for water and (1.4 ± 0.3) x $10^{-10} \frac{cm^2}{s}$ for ethanol.

To better understand the effect of surface and bulk fluorination on membrane separating properties, the diffusion coefficient estimates were used in conjunction with the pervaporation data in Figure 8 and the suggested model in Figure 9 to estimate the sorption and diffusion contributions

of pNB and pNBF8 in membrane selectivity. Figure S12 displays how the model in Figure 9 can be methodically stepped through in the rest of this section to give estimates for the sorption and diffusion components of selectivity. All reported errors in this section were calculated through standard propagation of errors rules:

$$\frac{\Delta z}{z} = \sqrt{\left(\frac{\Delta x}{x}\right)^2 + \left(\frac{\Delta y}{y}\right)^2} \quad (S9)$$

where $\Delta x, \Delta y,$ and $\Delta z$ are the standard deviations of $x$, $y$, and $z$ when $z = \frac{x}{y}$.

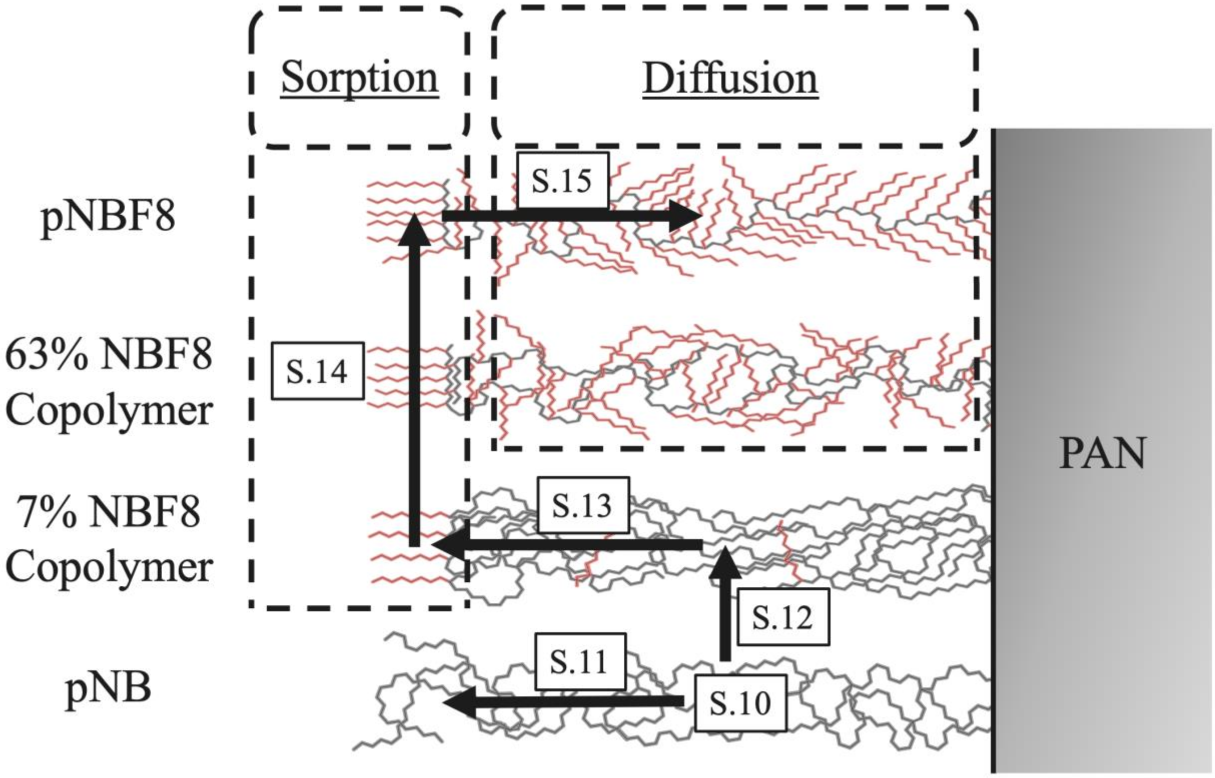


**Figure S13.** Calculation steps through Figure 9 to obtain Table 2 values by starting at the diffusion component of selectivity of pNB. Figure 9, and hence Figure S12, illustrates the relative concentrations of perfluorooctane chains for various p(NB-co-NBF8) films where hydrocarbon chains are in gray and fluorocarbon chains are in red. The areas of the fluorinated films that are expected to affect sorption and diffusion are denoted with dashed boxes.

The diffusion ratio for pNB was calculated by dividing the diffusion coefficients obtained from the kinetic curves of desorption:

$$\frac{D_{H_2O,\,pNB}}{D_{EtOH,pNB}} = \frac{(2.3 \pm 0.4)\ x\ 10^{-10}\,\frac{cm^2}{s}}{(1.4 \pm 0.3)\ x\ 10^{-10}\,\frac{cm^2}{s}} = 1.6 \pm 0.4 \quad (S10)$$

Taking the selectivity of pNB and dividing it by the diffusion component of pNB gives the sorption component of pNB:

$$\frac{S_{H_2O,pNB}}{S_{EtOH,pNB}} = \frac{\alpha_{pNB}}{\frac{D_{H_2O,\,pNB}}{D_{EtOH,pNB}}} = \frac{2.6 \pm 0.4}{1.6 \pm 0.4} = 1.6 \pm 0.5 \quad (S11)$$

Since the amount of fluorocarbon outside the surface region in the 7% NBF8 copolymer is minimal, it is assumed that the diffusion component of pNB is equivalent to that of the 7% NBF8 copolymer.

$$\frac{D_{H_2O,\,pNB}}{D_{EtOH,pNB}} = \frac{D_{H_2O,\,7\%\,NBF8\,Copolymer}}{D_{EtOH,7\%\,NBF8\,Copolymer}} = 1.6 \pm 0.4 \quad (S12)$$

The selectivity and diffusion component of the 7% NBF8 copolymer are then used to calculate the sorption component of the 7% NBF8 copolymer:

$$\frac{S_{H_2O,\,7\%\,NBF8\,Copolymer}}{S_{EtOH,7\%\,NBF8\,Copolymer}} = \frac{\alpha_{7\%\,NBF8\,Copolymer}}{\frac{D_{H_2O,\,7\%\,NBF8\,Copolymer}}{D_{EtOH,7\%\,NBF8\,Copolymer}}} = \frac{29 \pm 6}{1.6 \pm 0.4} = 18 \pm 6 \quad (S13)$$

Contact angle data suggests that the outer surface that would influence sorption is -$CF_3$ dominated in both the 7% NBF8 copolymer and the pNBF8; therefore, sorption components are expected to be the same between the polymer films.

$$\frac{S_{H_2O,\,7\%\,NBF8\,Copolymer}}{S_{EtOH,7\%\,NBF8\,Copolymer}} = \frac{S_{H_2O,\,pNBF8}}{S_{EtOH,pNBF8}} = 18 \pm 6 \quad (S14)$$

The selectivity and sorption component of the 7% NBF8 copolymer are then used to calculate the diffusion component of the pNBF8.

$$\frac{D_{H_2O,\,pNBF8}}{D_{EtOH,pNBF8}} = \frac{\alpha_{pNBF8}}{\frac{S_{H_2O,\,pNBF8}}{S_{EtOH,pNBF8}}} = \frac{155 \pm 86}{18 \pm 6} = 9 \pm 6 \qquad (S15)$$

**References**


(1) Autenrieth, B.; Schrock, R. R. Stereospecific Ring-Opening Metathesis Polymerization (ROMP) of Norbornene and Tetracyclododecene by Mo and W Initiators. *Macromolecules* **2015**, *48* (8), 2493–2503. https://doi.org/10.1021/acs.macromol.5b00161.

(2) Stoyanov, N. S.; Ramchandani, N.; Lemal, D. M. Functionalization of Saturated Fluorocarbons. *Tetrahedron Lett.* **1999**, *40* (36), 6549–6552. https://doi.org/10.1016/S0040-4039(99)01312-X.

(3) Burdeniuc, J.; Crabtree, R. H. Catalytic Photodefluorination of Perfluoroalkanes to Perfluoroalkenes with a Ferrocene Photosensitizer. *Organometallics* **1998**, *17* (8), 1582–1586. https://doi.org/10.1021/om970871q.

(4) Sandford, G. Perfluoroalkanes. *Tetrahedron* **2003**, *59* (4), 437–454. https://doi.org/10.1016/S0040-4020(02)01568-5.

(5) Parkerson, Z. J.; Prozorovska, L.; Vasuta, M. P.; Oddo, T. D.; Jennings, G. K. Simultaneous Spin Coating and Ring-Opening Metathesis Polymerization for the Rapid Synthesis of Polymer Films. *ACS Appl. Mater. Interfaces* **2024**, *16* (13), 16754–16766. https://doi.org/10.1021/acsami.4c00211.

(6) Fox, H. W.; Zisman, W. A. The Spreading of Liquids on Low Energy Surfaces. I. Polytetrafluoroethylene. *J. Colloid Sci.* **1950**, *5* (6), 514–531. https://doi.org/10.1016/0095-8522(50)90044-4.

(7) Owens, D. K.; Wendt, R. C. Estimation of the Surface Free Energy of Polymers. *J. Appl. Polym. Sci.* **1969**, *13* (8), 1741–1747. https://doi.org/10.1002/app.1969.070130815.

(8) Brantley, E. L.; Jennings, G. K. Fluorinated Polymer Films from Acylation of ATRP Surface-Initiated Poly(Hydroxyethyl Methacrylate). *Macromolecules* **2004**, *37* (4), 1476–1483. https://doi.org/10.1021/ma035471v.

(9) Seehof, N.; Grutke, S.; Risse, W. Selective Reaction with Exo-Isomers in Ring-Opening Olefin Metathesis Polymerization (ROMP) of Fluoroalkyl-Substituted Norbornene Derivatives. *Macromolecules* **1993**, *26* (4), 695–700. https://doi.org/10.1021/ma00056a021.

(10) Seah, M. P.; Dench, W. A. Quantitative Electron Spectroscopy of Surfaces: A Standard Data Base for Electron Inelastic Mean Free Paths in Solids. *Surf. Interface Anal.* **1979**, *1* (1), 2–11. https://doi.org/10.1002/sia.740010103.

(11) Pintori, G.; Cattaruzza, E. XPS/ESCA on Glass Surfaces: A Useful Tool for Ancient and Modern Materials. *Opt. Mater. X* **2022**, *13*, 100108. https://doi.org/10.1016/j.omx.2021.100108.

(12) Comyn, J. *Polymer Permeability,* Chapman & Hall, London, 1985.

(13) J. Crank, The Mathematics of Diffusion 2nd ed., Claredon Press, London, 1975.

(14) Pulyalina, A.; Polotskaya, G.; Goikhman, M.; Podeshvo, I.; Chernitsa, B.; Kocherbitov, V.; Toikka, A. Novel Approach to Determination of Sorption in Pervaporation Process: A Case Study of Isopropanol Dehydration by Polyamidoimideurea Membranes. *Sci. Rep.* **2017**, *7* (1), 8415. https://doi.org/10.1038/s41598-017-08420-0.
